# Forelimb movements evoked by optogenetic stimulation of the macaque motor cortex

**DOI:** 10.1038/s41467-020-16883-5

**Published:** 2020-06-26

**Authors:** Hidenori Watanabe, Hiromi Sano, Satomi Chiken, Kenta Kobayashi, Yuko Fukata, Masaki Fukata, Hajime Mushiake, Atsushi Nambu

**Affiliations:** 1 Department of Physiology, Tohoku University School of Medicine, Sendai, 980-8575 Japan; 2 Division of System Neurophysiology, National Institute for Physiological Sciences, Okazaki, 444-8585 Japan; 3 Department of Physiological Sciences, SOKENDAI (The Graduate University for Advanced Studies), Okazaki, 444-8585 Japan; 4 Section of Viral Vector Development, National Institute for Physiological Sciences, Okazaki, 444-8585 Japan; 5 Division of Membrane Physiology, National Institute for Physiological Sciences, Okazaki, 444-8787 Japan

**Keywords:** Neuroscience, Physiology

## Abstract

Optogenetics has become an indispensable tool for investigating brain functions. Although non-human primates are particularly useful models for understanding the functions and dysfunctions of the human brain, application of optogenetics to non-human primates is still limited. In the present study, we generate an effective adeno-associated viral vector serotype DJ to express channelrhodopsin-2 (ChR2) under the control of a strong ubiquitous CAG promoter and inject into the somatotopically identified forelimb region of the primary motor cortex in macaque monkeys. ChR2 is strongly expressed around the injection sites, and optogenetic intracortical microstimulation (oICMS) through a homemade optrode induces prominent cortical activity: Even single-pulse, short-duration oICMS evokes long-lasting repetitive firings of cortical neurons. In addition, oICMS elicits distinct forelimb movements and muscle activity, which are comparable to those elicited by conventional electrical ICMS. The present study removes obstacles to optogenetic manipulation of neuronal activity and behaviors in non-human primates.

## Abstract (web summary)

Non-human primates are useful models for understanding the human brain but application of optogenetics to non-human primates is challenging. The authors used optogenetic intracortical microstimulation in the primary motor cortex of macaques to elicit distinct forelimb movements and muscle activity.

## Introduction

Optogenetics is a technique to manipulate neuronal excitability by using genetically coded, light-gated ion channels or pumps (opsins), and light. Optogenetics is widely used and has become an indispensable tool for investigation of the functions of the nervous system^1–4^. This method has several advantages over classical tools, such as electrical stimulation and pharmacological blockade, because it can excite or inhibit a specific population of neurons with a high time resolution. Optogenetics has been successfully used to modulate behaviors in rodents^5–7^.

However, its application in non-human primates is still rather limited. Optogenetic stimulation has been attempted to induce and/or modulate body and eye movements, which can be easily elicited by electrical intracortical microstimulation (eICMS) with weak currents. Eye movements can be successfully modulated by optogenetic activation or inactivation of the cerebral cortex^8–12^. On the other hand, optogenetic stimulation of the motor cortices in monkeys failed to induce apparent body movements, although it evokes or modulates cortical activity^13,14^. This may be because opsins are not sufficiently expressed in neurons, and/or light does not effectively penetrate the monkey brain tissue^13,15^.

Inducing movements by optogenetics is a very important next step in non-human primate research. In the present study, we have overcome these drawbacks by injecting effective viral vectors into the identified primary motor cortex (M1) to express channelrhodopsin-2 (ChR2) and by using higher intensity light than previous studies through a homemade optrode that combines an optical fiber with recording and electrical stimulating electrodes. After these modifications, optogenetic intracortical microstimulation (oICMS) of the M1 successfully induces forelimb movements and muscle activity that are comparable to those induced by eICMS. The optrode also allows us to record neuronal activity evoked by oICMS and compare the effects of oICMS and eICMS.

## Results

### Gene transfer mediated by adeno-associated virus (AAV) vectors

To achieve the effective AAV vector-mediated gene transfer in the macaque brain, we compared different serotypes of AAV vectors, i.e., 2, 5, and DJ. First, electrophysiological mapping was carried out in the M1 to determine the sites for viral vector injections (Supplementary Fig. 1a). Then, we injected each serotype of AAV vector containing the ubiquitous CAG promoter and the enhanced green fluorescent protein (EGFP) transgene (1.5 × 10^12^ viral genome (vg)/ml for AAV2, AAV5, and AAV-DJ; 1 µl/site, 2–5 sites) into the M1 of two monkeys (Supplementary Table 1). Two to four weeks after AAV injection, when the transgene was expected to be expressed, we processed the brain sections and observed EGFP under a fluorescent microscope with the same exposure for the three different serotypes. Each injection site was separated at least 2 mm (for the same serotype) or 3 mm (for the different serotypes) (Supplementary Fig. 1a), and EGFP expression transduced by each serotype of viral vector in the brain sections was largely separated. However, a few EGFP-positive axons overlapped (but no cell bodies), so EGFP-positive areas could not be completely separated. The fluorescence signals were most conspicuous around the injection sites of the AAV-DJ vector (Supplementary Fig. 1b). The areas and number of cells transduced by the AAV-DJ vector were larger than those transduced by AAV2 and AAV5 (Supplementary Fig. 1c). The intensity of fluorescence signals at the single-neuron level by the AAV-DJ vector was higher than that by AAV2 and AAV5 (Supplementary Fig. 2a). Observing expression of NeuN, parvalbumin (PV), and glial fibrillary acidic protein (GFAP) among all EGFP-positive cells (Supplementary Fig. 2b, c) showed that the major cell type expressing EGFP was NeuN-positive neurons, and that glial cells expressing EGFP were a small population (Supplementary Fig. 2b). The projection sites of the M1 were also examined. The AAV-DJ vector induced EGFP expression at putative axon terminals in the putamen and at axons presumably in the lateral corticospinal tract of the spinal cord, but no retrogradely labeled cells were found in the thalamus (Supplementary Fig. 2d). In addition, the EGFP protein level induced by the AAV-DJ vector in the mouse brain was higher than that induced by AAV2 and AAV5 (Supplementary Fig. 3).

**Fig. 1:**
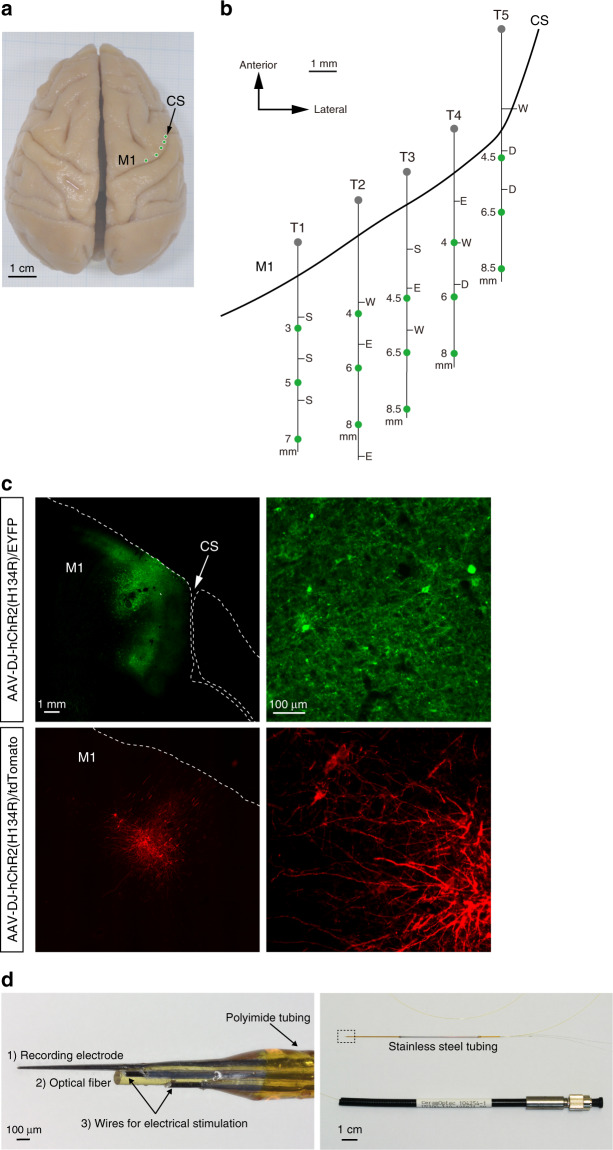
Mapping of the motor cortex expression of ChR2, and construction of the optrode. **a** Top view of the whole brain of a macaque (Monkey HJ). Green circles indicate penetration sites for AAV injections. CS central sulcus, M1 primary motor cortex. **b** Electrophysiological mapping of the right M1 (Monkey HJ). Somatotopic arrangement in the anterior bank of the CS is shown along with depths from the cortical surface. Each letter indicates a somatotopic body part: D digit, E elbow, S shoulder, W wrist. The AAV vector was injected in the sites indicated by the green circles. **c** Expression of hChR2(H134R)/EYFP and of hChR2(H134R)/tdTomato mediated by AAV-DJ vectors in the M1 shown in frontal sections (Monkey NR). These experiments were repeated three times independently (Supplementary Table 1), with similar results obtained. **d** Photograph of the tip (left) and whole view (right) of the optrode. (1) A glass-coated tungsten electrode for recording, (2) an optical fiber for optical stimulation and (3) a pair of tungsten wires for bipolar electrical stimulation were bundled and inserted into polyimide tubing (left). The base of the optrode was covered with stainless steel tubing (right). The other end of the optical fiber was jacketed with a protective tubing with a length of around 15 cm and had a fiber-optic connector. The rectangular area in the right photograph is enlarged in the left.

Therefore, we chose the AAV-DJ vector and generated a vector containing ChR2 with a point mutation [hChR2(H134R)] and a strong ubiquitous promoter CAG, i.e., AAV-DJ-CAG-hChR2(H134R)/EYFP or AAV-DJ-CAG-hChR2(H134R)/tdTomato. We first electrophysiologically mapped the anterior bank of the central sulcus and identified layer 5 of the forelimb region of the M1 by large amplitude of neuronal spikes and low motor threshold for eICMS (Fig. 1a, b). Based on the mapping, we injected the either one of them into these identified sites (8–15 sites, 1 µl/site; Supplementary Table 1). Three weeks after AAV injection when hChR2 was expected to be expressed, recording and stimulating experiments were started. Postmortem histological examinations showed distinct fluorescent signals of hChR2(H134R)/EYFP or hChR2(H134R)/tdTomato around the injection sites (Fig. 1c): The area covered by hChR2(H134R)/EYFP signals (18.0 mm^2^/µl injected, Monkey NR, left M1) was larger than that by the tdTomato-containing vector (4.2 mm^2^/µl injected, Monkey NR, right M1).

### Neuronal activity in the M1 evoked by optogenetic stimulation

We made an optrode by bundling (1) a glass-coated tungsten electrode for recording, (2) an optical fiber for optical stimulation, and (3) a pair of tungsten wires for bipolar electrical stimulation (Fig. 1d, see Methods for details). This optrode enabled us to record neuronal activity induced by optical and electrical stimulation. To examine responses of cortical neurons to optical stimulation, we inserted the optrode into the forelimb regions of the M1 where the AAV-hChR2 vector was injected (Supplementary Table 1). Then, oICMS was delivered through the optical fiber, which was connected to a solid-state laser source (473 nm blue laser), and neuronal activity was recorded through the recording electrode. Although electrical stimulation usually produces large stimulus artifacts and hinders recording of neuronal responses just after stimulation, oICMS induced negligible stimulus artifacts and therefore enabled us to analyze evoked cortical activity precisely.

**Fig. 2:**
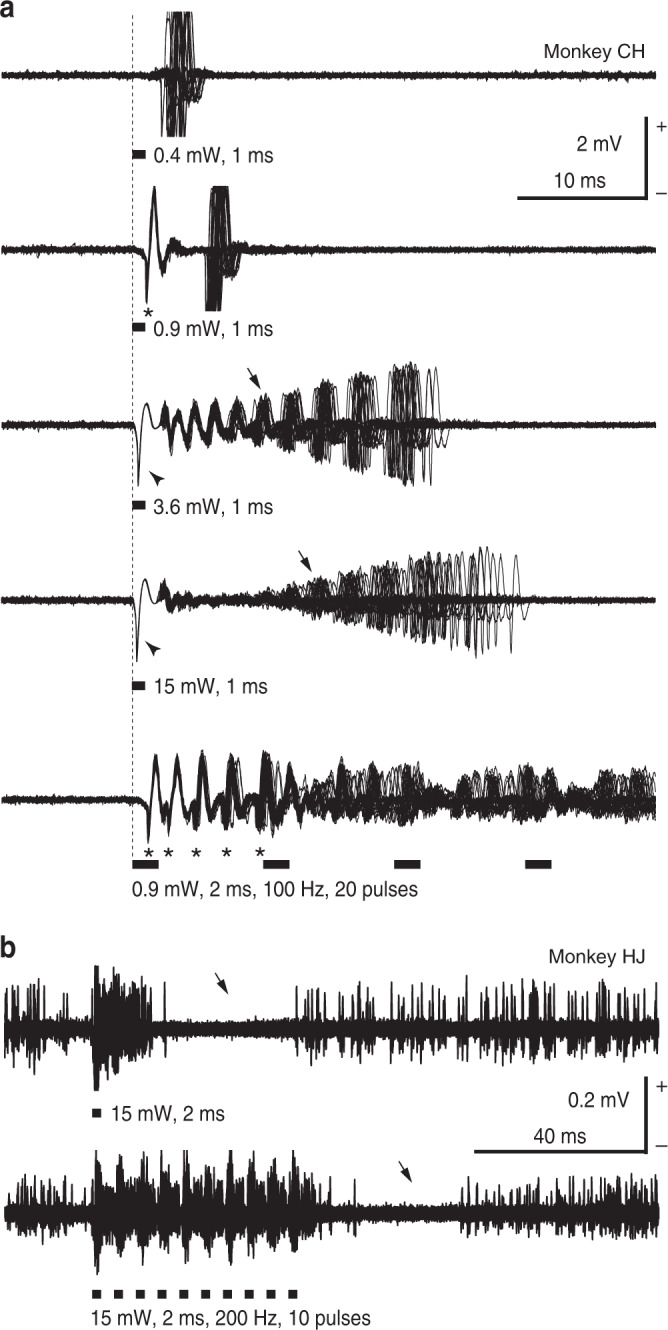
Raw traces of M1 neuronal activity evoked by optogenetic intracortical microstimulation (oICMS). **a** An example of M1 neuronal activity induced by various intensities of single-pulse (0.4, 0.9, 3.6, 15 mW strength corresponding to 51, 115, 458, and 1910 mW/mm^2^, respectively, 1 ms pulse duration) and repetitive (0.9 mW corresponding to 115 mW/mm^2^, 2 ms, 100 Hz, 20 pulses) oICMS. The neuronal activity from one and the same neuron probably located in layer 5 was recorded, and 30 traces are overlaid. A vertical dashed line and thick black horizontal lines indicate the beginning and duration of light illumination, respectively. *Action potentials at a short and constant latency that were most likely evoked directly; arrowheads, deflections from the baseline that were composed of population spikes of multiple neurons and/or local field potentials occurring at the same time; arrows, deteriorated action potentials that were truncated due to long-lasting depolarization. Note that no spikes were observed before stimulation, suggesting that the recording neuron was not injured by the optrode. **b** Another example of M1 neuronal activity induced by single-pulse (15 mW corresponding to 1910 mW/mm^2^, 2 ms) and repetitive (15 mW, 2 ms, 200 Hz, 10 pulses) oICMS shown on a long time scale. Multi-unit activity was recorded probably in layer 5. Excitation induced by oICMS was followed by inhibition (arrows).

Both single-pulse and repetitive oICMS within 1 mm from the injection sites of the viral vector successfully induced neuronal activity in all five monkeys examined (Fig. 2, Supplementary Table 2). Figure 2a shows a typical example of recordings from a single neuron. Low-intensity oICMS less than 1 mW (corresponding to 127 mW/mm^2^) was sufficient to evoke spikes in cortical neurons (Fig. 2a, 0.4 mW strength corresponding to 51 mW/mm^2^, 1 ms pulse duration), suggesting a high level of hChR2 expression. The minimum intensity and duration required to evoke cortical activity were 0.4 mW and 0.1 ms, respectively. Such low-intensity stimulation just above the threshold evoked an action potential at a latency of 2–4 ms (Fig. 2a, 0.4 mW, 1 ms) with time jitter. A slightly higher intensity oICMS evoked an action potential at a short and constant latency (less than 1.5 ms; * in Fig. 2a, 0.9 mW corresponding to 115 mW/mm^2^, 1 ms) followed by a spike with time jitter. Cortical stimulation usually induces both directly evoked excitation and synaptically induced excitation via axon collaterals of cortical neurons^16–18^. Action potentials at a short and constant latency and the following spikes with time jitter may correspond to directly evoked and synaptically induced excitation, respectively. Longer and repetitive oICMS induced repetitive directly evoked spikes (* in the bottom trace of Fig. 2a, 0.9 mW, 2 ms). Even higher intensity oICMS often induced a large deflection from the baseline (arrowheads in Fig. 2a, 3.6 mW, 15 mW, corresponding to 458 and 1910 mW/mm^2^, 1 ms). The deflection is unlikely an artifact, because (1) it was not induced by a 589 nm yellow laser (Supplementary Fig. 4a), and (2) it was not observed at non-injection sites (Supplementary Fig. 4b). Instead, the deflection seems to be composed of population spikes of multiple neurons and/or local field potentials that occurred at the same time. The deflection started just after the onset of oICMS, corresponding to an extremely fast rising time of ChR2-induced photocurrents with no visible delay^19^. The latency of deflection peaks was typically <1.0 ms, and more intense stimulation shortened the latency (Fig. 2a, 3.6 mW, 15 mW, 1 ms). The deflection was immediately followed by repetitive action potentials with a small amplitude that gradually recovered (arrows in Fig. 2a, 3.6 mW, 15 mW, 1 ms). The truncated action potentials that follow the stimulation could be explained by depolarization blocks induced by large depolarization in cortical neurons that last long after the cession of light illumination (up to 10 ms)^20^. Such truncated action potentials were observed at 55% (104/190 sites) of the sites where oICMS induced excitation. These complex responses evoked by oICMS were also observed in eICMS (Supplementary Fig. 5a). Both oICMS and eICMS induced excitation at a distance of 2.2–3.4 mm from the stimulation site (Supplementary Fig. 5b), suggesting that they both transsynaptically activated neurons in certain areas.

Figure 2b shows a typical example of multi-unit activity. The excitation evoked by single-pulse and repetitive oICMS was commonly followed by inhibition of firings (arrows in Fig. 2b; 198/359 recording neurons, 55%). One possible origin of the inhibition is inhibitory cortical interneurons activated synaptically by hChR2-expressing neurons or directly by light, although PV-positive inhibitory interneurons were less labeled with EGFP (Supplementary Fig. 2b). Alternatively, the inhibition may be caused by slow or medium afterhyperpolarization following high-frequency repetitive firings^21^. Figure 2b also shows that action potentials followed each stimulus pulse at 200 Hz (Fig. 2b, 15 mW corresponding to 1910 mW/mm^2^, 2 ms, 200 Hz, 10 pulses). The data in Fig. 2a, b indicate that oICMS effectively activates M1 neurons.

### Forelimb movements evoked by optogenetic stimulation

Next, we examined whether the oICMS-evoked M1 activity observed above could induce body movements. The tip of the optrode was placed in the forelimb region of the M1 where large neuronal responses were evoked by oICMS. Repetitive oICMS (1.8–15 mW, corresponding to 229–1910 mW/mm^2^, 1–2 ms pulse duration, 100–333 Hz, 10–20 pulses) clearly induced short-duration phasic movements in the contralateral forelimb of all five monkeys examined (Supplementary Table 2). Supplementary Movie 1 shows such examples: finger extension, muscle twitching in the forearm, and shoulder elevation. These movements were similar to those evoked by conventional eICMS. oICMS and eICMS shared the following features: (1) they elicited movements in the same parts of the forelimb in concert with the somatotopic map obtained in M1 mapping for vector injection, (2) both single-pulse and repetitive oICMS/eICMS induced movements, and the repetitive one was more effective, (3) the amplitude of movements evoked by oICMS was comparable to that evoked by eICMS, and (4) low-intensity oICMS/eICMS just above the motor threshold typically evoked confined movements, such as single-joint movements or muscle twitches, whereas higher intensity oICMS/eICMS induced large movements of multiple joints and body parts.

### Muscle activity evoked by optogenetic stimulation

**Fig. 3:**
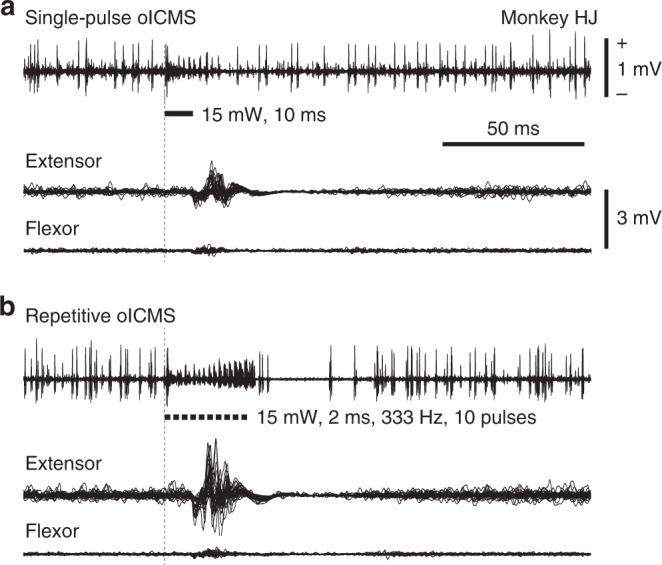
Simultaneously recorded M1 and muscle activity evoked by oICMS. **a**, **b** M1 activity (top trace) and electromyograms (EMG; bottom two traces) induced by single-pulse (**a**, 15 mW strength corresponding to 1910 mW/mm^2^, 10 ms pulse duration) and repetitive (**b**, 15 mW, 2 ms, 333 Hz, 10 pulses) oICMS. M1 neuronal activity was recorded probably from neurons in layer 5. EMG was recorded from the extensor carpi radialis longus (Extensor) and flexor carpi radialis (Flexor) muscles. Vertical dashed lines and thick black horizontal lines indicate the beginning and duration of light illumination, respectively. Calibration in **a** is applicable to **b**.

**Fig. 4:**
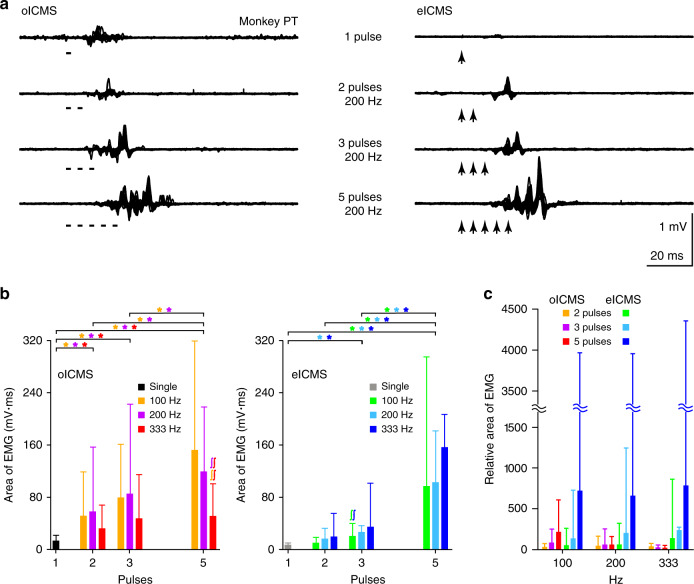
Muscle activity evoked by oICMS and electrical ICMS (eICMS). **a** Raw EMG recorded from the flexor carpi radialis muscle. One, two, three, and five stimulus pulses of oICMS (left, 15 mW strength corresponding to 1910 mW/mm^2^, 2 ms pulse duration, 200 Hz) and eICMS (right, 50 µA strength, 0.2 ms pulse duration, 200 Hz) were applied to the same site. **b** The area of EMG evoked by oICMS (left, 15 mW corresponding to 1910 mW/mm^2^, 2 ms) and eICMS (right, 40–65 µA, 0.2 ms) with one, two, three, and five pulses at different frequencies. The EMG data were averaged across stimulating sites (33 and 19 sites for oICMS and 19 and 20 sites for eICMS in Monkeys HJ and PT, respectively). Bar graphs represent means and SDs. Different colors indicate different frequencies (single-pulse, 100, 200, and 333 Hz). *p < 0.05, significantly different between stimulus pulses in the frequency indicated by the color; ^∫^*p* < 0.05, significantly different from each other between stimulus frequency indicated by the color (Friedman test with Dunn’s post hoc test). **c** The relative area of EMG evoked by repetitive oICMS (warm colors) and eICMS (cold colors) with two, three, and five pulses (indicated by different colors) at different frequencies (100, 200, and 333 Hz). The same datasets as in **b** were used, and the area of EMG was normalized to that evoked by single-pulse oICMS/eICMS. Bar graphs represent means and SDs.

To examine the relationship between M1 activity and body movements, we simultaneously recorded M1 activity with the optrode and electromyogram (EMG) of the forelimb with surface dish electrodes in four monkeys (Supplementary Table 2). Both single-pulse and repetitive oICMS induced M1 activity (Fig. 3a, b, top traces), and shapes of spikes during oICMS were truncated by strong depolarization as seen in Fig. 2a. They also induced muscle activity of extensor and flexor muscles (Fig. 3a, b, bottom two traces). The mean threshold to elicit muscle activity by single-pulse oICMS (10 ms duration) was 10.5 ± 6.3 mW (1337 ± 802 mW/mm^2^), and the range was 0.4–15 mW (51–1910 mW/mm^2^). Higher intensity of oICMS was more effective than lower intensity as in the case of eICMS. Repetitive oICMS evoked stronger M1 and muscle activity than single-pulse oICMS (Figs. 3 and 4a, left) as in the case of repetitive eICMS (Fig. 4a, right). The latency of muscle activity evoked by oICMS was 14.0 ± 3.9 ms (*n* = 52 sites; 15 mW corresponding to 1910 mW/mm^2^, 2 ms, 200 Hz, 5 pulses), which was comparable to that induced by eICMS (12.8 ± 3.2 ms, *n* = 39 sites; 40–65 µA, 0.2 ms, 333 Hz, 5 pulses).

To examine the effects of stimulus pulse numbers and frequencies on muscle activity, we compared the area and peak amplitude (Fig. 4, Supplementary Fig. 6) of EMG evoked by oICMS (52 sites) and eICMS (39 sites) with different numbers of pulses (one, two, three, and five pulses) and frequencies (100, 200, and 333 Hz). Five pulses of oICMS and eICMS showed comparable area and peak amplitude of EMG (Fig. 4b, Supplementary Fig. 6a, b). When the number of stimulus pulses was decreased, the area and peak amplitude obtained by oICMS showed a modest decrease compared to eICMS.

Regarding the area of EMG, two and three pulses of oICMS at 100 and 200 Hz (Fig. 4b, left) induced significantly smaller EMG than five pulses of oICMS (two pulses, *p* < 0.0001 at 100 and 200 Hz; three pulses, *p* = 0.0002 at 100 Hz, *p* = 0.013 at 200 Hz; Friedman test with Dunn’s post hoc test), but no significant differences at 333 Hz (*p* > 0.05). One pulse of oICMS induced significantly smaller EMG than two, three, and five pulses of oICMS (two pulses, *p* < 0.0001 at 100 and 200 Hz, *p* = 0.009 at 333 Hz; three pulses, *p* < 0.0001 at 100 and 200 Hz, *p* = 0.0002 at 333 Hz; five pulses, *p* < 0.0001 at 100, 200, and 333 Hz). On the other hand, the area by one, two, and three pulses of eICMS (Fig. 4b, right) was all significantly smaller than that by five pulses of eICMS at 100, 200, and 333 Hz (one and two pulses, *p* < 0.0001 at 100, 200, and 333 Hz; three pulses, *p* < 0.0001 at 100 and 200 Hz, *p* = 0.025 at 333 Hz). The area by one and two pulses of eICMS (*p* > 0.05) and that by two and three pulses (*p* > 0.05) were not different from each other, whereas that by one pulse was significantly smaller than that by three pulses at 200 and 333 Hz (*p* < 0.0001 at 200 and 333 Hz). These results suggest that repetitive oICMS as well as eICMS induces muscle activity more effectively than single-pulse oICMS and eICMS. Concerning the frequency of repetitive stimulation, 333 Hz-stimulation was less effective in five pulses of oICMS (Fig. 4b, left): The area of EMG by 333 Hz-oICMS was significantly smaller than that by 100 and 200 Hz-oICMS (*p* < 0.0001), suggesting that the effects of oICMS were saturated and became smaller at 333 Hz. On the other hand, the effects of different frequencies were smaller in eICMS: The area of EMG evoked by 100, 200, and 333 Hz-eICMS was not different from each other (Fig. 4b, right), except that three pulses of eICMS at 100 Hz induced smaller EMG than that at 333 Hz (*p* = 0.018).

The different effects by oICMS and eICMS were also observed in the relative area of EMG and the semilog plots of the area of EMG evoked by repetitive oICMS/eICMS (Fig. 4c, Supplementary Fig. 6a). The relative area of EMG evoked by oICMS increased as the numbers of stimulus pulses increased at 100 Hz, but did not at 200 and 300 Hz (Fig. 4c). On the other hand, the relative area of EMG by eICMS increased as the number of stimulus pulses increased at all frequencies. The semilog plots indicate that the area of EMG evoked by eICMS increased more rapidly than that by oICMS as the numbers of stimulus pulses increased in a nearly exponential manner (Supplementary Fig. 6a). These results suggest that oICMS shows less temporal summation than eICMS. The peak amplitude of EMG showed similar tendency (Supplementary Fig. 6b).

## Discussion

Introduction of optogenetics has revolutionized stimulation methods^1–4^, and provided several advantages over electrical stimulation. Optogenetics can selectively stimulate a specific population of neurons or specific pathways by labeling them with light-sensitive opsins using transgenic techniques and/or viral vectors. So far, most optogenetic studies have been performed in rodents and reported photo-induced neuronal and behavioral changes. In the early optogenetic studies, optical stimulation of the motor cortex in rodents expressing ChR2 induced apparent whisker deflection or limb movements^6,7,22,23^. Despite these remarkable results in rodents, optogenetic stimulation of the sensorimotor cortex in macaque monkeys using ChR2 failed to modulate arm and hand movements^13^. In another study, although gamma oscillations were induced in the macaque motor cortex by optogenetic stimulation using a ChR2 variant, no clear behavioral modulation was observed^14^. Transduction of enhanced NpHR (eNpHR) into the macaque M1 hand-arm region caused suppression of M1 neuronal activity, but no obvious modulation of reaching and grasping behaviors was found^13,24^. On the other hand, optogenetic stimulation of the primary visual cortex^8^, the lateral intraparietal area^9^, the projection from the frontal eye field to the superior colliculus^10^, and Purkinje cells in the cerebellum^25^ modulated saccadic eye movements in macaque monkeys. Optogenetic approaches were also applied to subcortical regions and modulated neuronal activity^26–29^.

In the current study, we showed that oICMS in the M1 of macaque monkeys evoked clear forelimb movements that resembled movements evoked by eICMS (Figs. 3 and 4, Supplementary Movie 1, Supplementary Table 2). The key to our success in inducing obvious movements by optogenetics in macaque monkeys was a winning combination of the following: (1) A highly effective AAV serotype DJ and a strong ubiquitous promoter CAG enabled potent gene transfer of hChR2 into neurons, (2) The AAV vector was injected into somatotopically identified regions of the M1, (3) Our optrode could identify layer 5 cortical neurons expressing hChR2 and effectively illuminate them, and (4) Intensity of light (51–1910 mW/mm^2^) used for illumination was stronger than that in previous studies targeting the M1 (3–255 mW/mm^2^)^12,13,15^. Actually, oICMS changed neuronal activity more effectively (75% of cortical sites examined, Supplementary Table 2) than in previous reports (27.4–68.2% of neurons examined)^13,14^.

AAV vectors are the most widely applied tools in optogenetics and have been successfully used to deliver opsins into the mouse, rat, and non-human primate brains. Previous studies evaluated transduction efficiency of different serotypes of AAV vectors, i.e., 1, 2, 5, 8, and 9, in the cortex of macaque monkeys^30,31^. An AAV-DJ vector was created from DNA shuffling of eight AAV serotypes to mediate efficient gene transfer^32^ and showed high transduction effectiveness and neuronal tropism in the monkey putamen^33^. In the present study, in the macaque M1, we compared AAV2, AAV5, and AAV-DJ vectors expressing EGFP under the control of a ubiquitous CAG promoter that is commonly used in rodents. EGFP was successfully transduced by all AAV serotypes we injected, and the AAV-DJ vector showed the highest fluorescence intensity, the largest transduced area and number of cells, and the highest protein expression (Supplementary Figs. 1, 2, and 3). As an opsin for optical stimulation, we used a ChR2 variant, H134R, which was developed as a kinetic opsin variant in an early optogenetic study^4^ and obtained obvious behavioral modulation among many ChR variants developed. Thus, we prepared AAV-DJ vectors carrying CAG-hChR2(H134R)/EYFP or CAG-hChR2(H134R)/tdTomato to improve the expression levels of hChR2. In addition, we carefully performed electrophysiological mapping of the M1 and identified layer 5 of the forelimb region where large amplitude of neuronal spikes and eICMS-evoked forelimb movements were clearly observed (Fig. 1a, b). We then injected the AAV vector into the identified region. Expression of hChR2/EYFP and hChR2/tdTomato was observed in the areas including layer 5 of the M1 (Fig. 1c).

In the present study, we used high intensity of light (51–1910 mW/mm^2^) compared to previous studies. Previous studies targeting M1 cortical neurons expressing ChR2 used weaker light (3–255 mW/mm^2^)^13,14^ and could not affect behaviors. On the other hand, eye movements were successfully modulated by higher intensity light. Optogenetic inactivation of superior collicular (SC) neurons expressing Archaerhodopsin-T using green light (650–1600 mW/mm^2^) shifted saccadic end point, reduced peak velocity, and increased latency^12^. Optogenetic activation of ChR2-expressing terminals in the SC using blue light (<1100 mW/mm^2^)^10^, excited SC neuron, and evoked saccadic eye movements. Thus, we decided to use higher intensity light in this study. Such intensity of light seems to have no damage to the cortical neurons because (1) cortical neurons could be activated repeatedly by oICMS without any injury discharges, (2) neuronal and muscle activity could be induced by oICMS at the same site with the same threshold in different days, and (3) histological examination showed no apparent damage in the cortex.

We compared body movements and EMG evoked by oICMS and eICMS. Forelimb movements induced by oICMS were comparable to those induced by eICMS (Figs. 3 and 4, Supplementary Movie 1). Sometimes, we used only oICMS without eICMS, and observed evoked movements by oICMS. We also examined motor threshold of oICMS before and after eICMS, and did not see any threshold changes, suggesting no priming effects of eICMS on oICMS. The latency of muscle activity evoked by oICMS (14.0 ± 3.9 ms) was in the same range as that evoked by eICMS in the present (12.8 ± 3.2 ms) and previous (8.8–10.1 ms)^34^ studies. Both oICMS and eICMS can induce neuronal and muscle activity effectively (Figs. 3 and 4, Supplementary Fig. 5), although different population would be stimulated. oICMS illuminates brain tissue in front of the optrode along its long axis. Based on the study by Ozden et al.^35^, 1 mW of light through an 200-µm optical fiber sheds 1 mW/mm^2^ light inside a spheroidal area, whose size is 1 mm in the axial direction and 0.3 mm in the radial direction (for details, see Fig. 4a in Ozden et al.^35^). Inside this area, neurons expressing ChR2 are expected to be activated. When the optrode is inserted into the anterior bank of the central sulcus, light can activate layer 5 pyramidal neurons over a wide area. On the other hand, eICMS may activate neurons inside a sphere around the electrode tip, the size of which increases with increasing current^17,18^: 10 and 100 µA currents activate neurons in a radius of 100 and 450 µm around the electrode tip. However, a recent study using two-photon calcium imaging indicated that eICMS might sparsely activate neurons around the electrode, sometimes as far as millimeters away^36^. In the present study, the effects of oICMS at 333 Hz were less effective than those at 100 and 200 Hz. This is probably due to photocycle kinetics of hChR2: fast channel opening, but relatively slow channel closing and slow recovering to the ground state^37,38^. Actually, during high-frequency optical stimulation, more than 100 Hz, stimulus pulses often fail in inducing action potentials in previous studies^39,40^. Further studies are needed to elucidate the mechanism of oICMS especially in comparison with eICMS.

In this paper, we applied oICMS to the somatotopically identified region of the M1 and evoked forelimb movements successfully in a similar manner to eICMS. Our current study has removed obstacles to evoking movements by oICMS and opened a new window to the application of optogenetics to motor physiology in non-human primates: selective manipulation of an optional population of neurons by using a region-specific promoter and/or pathway-specific labeling.

## Methods

### Animals

Seven adult Japanese monkeys of both sexes (*Macaca fuscata*, body weight 3.0–10.5 kg) were used in this study (Supplementary Table 1). Experiments were performed at National Institute for Physiological Sciences (NIPS; Monkeys CL, NR, HJ, PT, and MG) and Tohoku University (TU; Monkeys CH and HK). Each monkey was daily trained to sit quietly in a monkey chair. The experimental protocols were approved by the Institutional Animal Care and Use Committees of National Institutes of Natural Sciences and Tohoku University. All experiments were conducted according to the guidelines of the National Institutes of Health Guide for the Care and Use of Laboratory Animals.

### Surgery

Under general anesthesia with ketamine hydrochloride (5–8 mg/kg body weight, i.m.), xylazine hydrochloride (0.5 mg/kg, i.m.), and propofol (6–7 µg/ml, blood concentration) (NIPS); or with ketamine hydrochloride (10 mg/kg, i.m.), xylazine hydrochloride (0.5 mg/kg, i.m.), and isoflurane (1–1.5% in room air, 2 l/min) (TU), the monkey underwent surgery to fix its head painlessly to a stereotaxic frame that could be attached to a monkey chair as described previously^41,42^. After skin incisions, the skull was widely exposed, and the periosteum was completely removed. Small polyether ether ketone (PEEK) screws were attached to the skull as anchors. The exposed skull and screws were completely covered with transparent acrylic resin. Two PEEK pipes were mounted in parallel over the frontal and occipital regions for head fixation. All surgical procedures were performed under aseptic conditions. Arterial oxygen saturation and heart rate were monitored during the surgery. Depth of anesthesia was assessed by heart rate and body movements. Additional anesthetic agents were administrated when necessary. Antibiotics and analgesics (ketoprofen) were administered (i.m.) after the surgery.

### Preparation of viral vectors

AAV vectors were packaged using the AAV Helper Free Expression System (Cell Biolabs) as described previously^43^. The transfer plasmids, pAAV-CAG-EGFP and pAAV-CAG-hChR2(H134R)-EYFP, were constructed, and pAAV-CAG-hChR2(H134R)-tdTomato was obtained from Addgene. The packaging plasmids (pHelper and pAAV-2, pAAV-5, or pAAV-DJ) and transfer plasmid were co-transfected into HEK293T cells using the calcium phosphate method. Crude cell extracts containing AAV vector particles were purified by ultracentrifugation with CsCl, dialyzed, and concentrated with an Amicon 10 K MWCO filter (Merck). The copy numbers of the viral genome (vg) determined by qPCR were 1.5 × 10^12^ vg/ml (AAV2-CAG-EGFP, AAV5-CAG-EGFP, and AAV-DJ-CAG-EGFP), 4.0 × 10^12^ vg/ml (CAG-hChR2(H134R)/EYFP), and 4.3 × 10^12^ vg/ml (CAG-hChR2(H134R)/tdTomato), respectively.

### Mapping of the motor cortex and injection of viral vectors

After full recovery from the surgery, each monkey was seated quietly in a monkey chair with its head painlessly fixed in a stereotaxic frame. The skull over the central sulcus was removed under anesthesia with ketamine hydrochloride (10 mg/kg, i.m.) and xylazine hydrochloride (0.2–0.3 mg/ kg, i.m.) with local lidocaine or bupivacaine application. A rectangular plastic chamber was attached to cover the opening. The forelimb region of the M1 was identified by electrophysiological mapping^41,42,44,45^ (Fig.1a, b; Supplementary Fig. 1a). Briefly, a glass-coated Elgiloy-alloy microelectrode was inserted perpendicularly to the cortical surface. Extracellular neuronal activity was recorded, and responses to somatosensory stimuli were examined. Then, eICMS (monopolar stimulation, 12 cathodal pulses, <50 µA strength, 0.2 ms pulse duration, 333 Hz) was applied, and evoked movements were observed. We identified layer 5 of the forelimb region of the M1 by large amplitude of neuronal spikes and low motor threshold for eICMS to evoke movements.

Based on the mapping, AAV vectors carrying the CAG-EGFP (to Monkeys CL and NR), CAG-hChR2(H134R)/EYFP (to Monkeys NR, HJ, and PT), or CAG-hChR2(H134R)/tdTomato (to Monkeys CH, HK, and NR) transgene were injected into the forelimb region of the M1 (Supplementary Table 1). A glass micropipette was made of a 3-mm glass capillary by using a puller (PE-2, Narishige) and connected to a 25-µl Hamilton microsyringe by Teflon tubing (JT-10, Eicom). A tungsten wire was inserted into the glass micropipette to record neuronal activity. The glass micropipette, tubing, and a Hamilton microsyringe were filled with Fluorinert (FC-3283, Sumitomo 3M). The syringe was mechanically controlled by a syringe pump (UMP3, WPI). Viral vector solution was loaded from the micropipette. The glass micropipette was inserted into the M1 perpendicularly to the cortical surface through a small incision in the dura mater. Injection sites were selected in putative layer 5 by recording large amplitude of neuronal spikes through the wire in the glass micropipette. Then, the vector solution (1 µl at each site) was injected slowly (50 nl/min). The micropipette was left in place for an additional 10 min and then slowly withdrawn. AAV vectors were injected into 2–5 sites (each serotype carrying CAG-EGFP, Supplementary Fig. 1a) or 4–15 sites (CAG-hChR2(H134R)/EYFP or CAG-hChR2(H134R)/tdTomato, Fig. 1a, b) in each hemisphere (Supplementary Table 1).

### Construction of optrodes

An optrode with recording and stimulating electrodes was constructed in our laboratory from three major components (Fig. 1d, left): (1) a glass-coated tungsten electrode for recording (369-120610-00, 1 MΩ, Alpha Omega), (2) an optical fiber with a 100-µm diameter silica core (FC1P/UV100/110/125 P/BPGS-0.15M/1.5M, CeramOptec) for oICMS, and (3) a pair of 50-µm diameter tungsten wires for bipolar eICMS, which were coated with Teflon except at their tips (California Fine Wire). The optical fiber was attached to the recording electrode 800–1000 µm behind the tip of the recording electrode. The pair of tungsten wires for eICMS was attached to the optical fiber (the distance between wire tips, 300–400 µm). The three components were fixed together by applying small drops of standard cyanoacrylate glue and epoxy glue, and then inserted into polyimide tubing (Microlumen). The base of the optrode was covered with stainless steel tubing (Fig. 1d, right). The tip of the optrode was observed under a digital microscope (VHX-1000 and 7000, Keyence) (Fig. 1d, left). The other end of the optical fiber was jacketed with a protective tubing with a length of around 15 cm and had a fiber-optic connector (Fig. 1d, right). The optical fiber was connected to a solid-state laser source (473 nm, 100 or 200 mW power output; COME2-473-100LS or COME2-LB473/200, Lucir). Light power was measured in mW using a power and energy meter (Ophir Optronics) at a distance of 1 mm from the light-emitting tips and calculated as power per unit area (mW/mm^2^).

### Optogenetic stimulation and extracellular recording in the M1

Three weeks after vector injection when hChR2 was expected to be expressed, recording and stimulating experiments were started. Each monkey was seated in a monkey chair in awake conditions. Two stainless steel rods were inserted into PEEK pipes over its head and were fixed in a stereotaxic frame without any pain as described previously^41,42^. An optrode was mounted in a micromanipulator (MO-81-S, Narishige) and inserted into the M1 perpendicularly to the cortical surface through a small incision of the dura mater. Electrophysiological signals from the recording electrode were amplified (5000 times), filtered (300–3000 Hz), digitized at 50 kHz, and stored on a computer using LabView software (National Instruments). For oICMS, the optical fiber was connected to the solid-state laser source, which was controlled by a stimulator (SEN8201, Nihonkohden). Single-pulse (0.4–15 mW strength corresponding to 51–1910 mW/mm^2^, 0.1–10 ms pulse duration) or repetitive (0.4–15 mW strength, 0.1–2 ms pulse duration, 100–333 Hz, 2–50 pulses) oICMS was applied through the optical fiber at every 1.4 or 2.4 s. The stimulus frequency of repetitive oICMS followed that was commonly used in repetitive eICMS in previous studies^41,42^. For eICMS, the tungsten wires for bipolar stimulation were connected to an isolator (SS-202J, Nihonkohden) and a stimulator (SEN8201), and single-pulse (monophasic, 20–65 µA strength, 0.2 ms pulse duration) or repetitive (monophasic, 20–65 µA strength, 0.2 ms pulse duration, 100–333 Hz, 2–20 pulses) eICMS was applied through the bipolar stimulating electrodes at every 1.4 s.

### EMG recording

Body movements and EMG were observed when monkeys were at rest without any movements. Body movements evoked by oICMS or eICMS were video-recorded. The timing of oICMS/eICMS was indicated by the flash of LEDs set near the body parts recorded. When body movements were observed, a pair of surface dish electrodes (NE-05, Nihonkohden) were attached to the skin over the belly of the responsible muscle. Muscle activity was amplified (5000 times), filtered (50–3000 Hz), digitized at 50 kHz, and stored on a computer simultaneously with neuronal activity using LabView software. Although body parts in which movements and/or EMG were evoked depended on the cortical site of oICMS/eICMS, EMG of the extensor carpi radialis longus and flexor carpi radialis muscles was mainly recorded and used for further analyses.

### Histological examination

After collection of enough data (in the case of electrophysiological experiments) or 2–4 weeks after AAV injections (for evaluation of AAV vectors), monkeys were deeply anesthetized with sodium pentobarbital (50 mg/kg, i.v.) and perfused transcardially with 0.1 M phosphate buffer (PB, pH 7.3) followed by 10% formalin in 0.1 M PB, and 0.1 M PB containing 10% sucrose (Supplementary Table 1). The brains were removed and kept in 0.1 M PB containing 30% sucrose at 4 °C. Frontal sections (50 μm thick) were cut serially with a freezing microtome and collected in 0.01 M PBS. Every sixth sections were placed onto gelatine-coated glass slides, air dried, and mounted in Vectashield (Vector Laboratories). The remaining sections were used for immunohistochemistry to enhance fluorescence. Every sixth free-floating sections were incubated with primary antibodies against rabbit GFP (1:1000; Thermo Fisher Scientific) or rabbit DsRed (1:500; Takara Clontech) at 4 °C overnight, and then visualized with secondary antibodies conjugated with Alexa Fluor 488 or Alexa Fluor 594 (1:1000; Thermo Fisher Scientific). All fluorescent images were captured by a fluorescence microscope (BZ-X710 and BZ-X Viewer, Keyence).

### Data and statistical analyses

Recorded EMG was rectified, re-sampled at 2 kHz, and averaged for 30 stimulus trials using Igor software (WaveMetrics). The mean and SD values of EMG during the 100-ms period preceding the onset of stimulation were considered as the baseline activity. Changes in EMG in response to stimulation were judged significant if the EMG during at least six consecutive points (3 ms) reached a significant level of *p* < 0.01 (one-tailed *t*-test). The latency was defined as the time at which the first point exceeded this level. The responses were judged to end when six consecutive points fell below the significance level. The end point of the responses was determined as the time at which the last point exceeded this level. The area of EMG was defined as the area of averaged EMG during the significant response over the baseline. The relative area of EMG was defined as the ratio of the area of EMG to that evoked by single-pulse oICMS/eICMS. The peak amplitude of EMG was defined as the height of maximal peak in the averaged EMG. Data containing weak EMG, defined as small area (<20 mV ms) or amplitude (<2 mV) of EMG evoked by oICMS or eICMS (five pulses at 200 Hz) were excluded from further analyses. The area and peak amplitude of EMG were compared between different stimulus pulse numbers and between different stimulus frequencies by Friedman test with Dunn’s post hoc test. Significant level was set to *p* < 0.05. Statistical analyses including curve fitting were performed using Prism software (versions 6 and 7, GraphPad).

### Reporting summary

Further information on research design is available in the Nature Research Reporting Summary linked to this article.

## Supplementary information

Supplementary Information

Peer Review File

Supplementary Movie 1

Description of Additional Supplementary File

Reporting Summary

## Data Availability

The data that support the findings of this study are available from the corresponding author (nambu@nips.ac.jp) upon reasonable request. The Source data underlying Fig. 4b (Supplementary Fig 6a), c, Supplementary Figs. 1c, 2a, 2b, 3b, 6b can be found in a Source Data file. Source data are provided with this paper.

## Source data

Source data
