# Supplementary material for: Forelimb movements evoked by optogenetic stimulation of the macaque motor cortex
Source: Nat Commun. 2020 Jun 26;11:3253. doi: 10.1038/s41467-020-16883-5 (PMC7319997; doi:10.1038/s41467-020-16883-5)
Supplement: Supplementary file 1 — Supplementary Information [file 41467_2020_16883_MOESM1_ESM.pdf]

Supplementary Information

Forelimb movements evoked by optogenetic stimulation of the macaque motor cortex

Watanabe et al.

List of Contents

Supplementary Methods

Supplementary Tables 1 and 2

Supplementary Figures 1-7

Supplementary Methods

Histological examination and analysis. Every sixth free-floating sections of the monkey brain were incubated with primary antibodies against rabbit GFP (1:1000; Thermo Fisher Scientific), mouse NeuN (1:400; Merck), mouse parvalbumin (PV) (1:5000; Swant), and mouse glial fibrillary acidic protein (GFAP) (1:400; Sigma) at 4℃ overnight, and then visualized with secondary antibodies conjugated with Alexa Fluor 488 or Alexa Fluor 594 (1: 1000; Thermo Fisher Scientific). All fluorescent images were captured by a fluorescence microscope (BZ-X710 and BZ-X Viewer, Keyence). For measuring the extent of the areas covered by EGFP-positive cells transduced with AAV2 (examined in 8 sections), AAV5 (10 sections), or AAV-DJ (12 sections), and hChR2(H134R)/EYFP (13 sections) or hChR2(H134R)/tdTomato (6 sections) signals were manually surrounded and measured using ImageJ software (National Institutes of Health). For measuring the fluorescence intensity of EGFP, all fluorescent images were captured with same exposure, then individual EGFP positive cells in 500 μm × 500 μm region of interest (ROI) were surrounded manually, and the optical density was measured using ImageJ software in 8-12 ROIs. The fluorescence intensity by AAV2 injection was averaged over all cells and used as the reference value. The fluorescence intensity by AAV2, AAV5, and AAV-DJ injection was expressed as a ratio to the reference value. For counting of EGFP, NeuN, PV, and GFAP positive cells, 10-55 ROIs (500 μm × 500 μm) were examined manually using ImageJ software.

Quantitative western blotting. To compare AAV2, AAV5, and AAV-DJ vectors, we injected one of these vectors into the mouse brain and performed western blot analyses. Experiments were performed at National Institute for Physiological Sciences. The experimental protocols were approved by the Institutional Animal Care and Use Committee of National Institutes of Natural Sciences. AAV2, AAV5, or AAV-DJ vector carrying the CAG-EGFP transgene (4×10^{12} viral genome (vg)/ml, 0.5 μl/site, two sites) was injected into each hemisphere of the

mouse under general anesthesia with isoflurane (1.0-1.5%). Two weeks after AAV injection, mice were sacrificed by cervical dislocation, then the brain hemispheres were dissected and homogenized separately with 10-volume of buffer containing 20 mM Tris-HCl (pH 8.0), 1 mM EDTA, 320 mM sucrose, and 100 μg/ml phenylmethylsulfonyl fluoride. The protein concentration of individual homogenates was measured by BCA protein assay (Thermo Fisher Scientific), and the homogenates were diluted to 1 mg/ml in SDS-PAGE sample buffer. Individual homogenates (15 μg each) were subjected to western blotting with anti-GFP and anti-β-catenin (as an internal control) antibodies. Anti-GFP rabbit polyclonal antibody was raised against GST-GFP and affinity-purified. Anti-β-catenin mouse monoclonal antibody (clone, 14/beta-catenin) was purchased from BD Biosciences. For quantitative western blotting, chemical luminescent signals were detected with the FUSION Solo system (Vilber-Lourmat) and analyzed with the FUSION software.

Supplementary Tables

Monkey	Male/ female	BW (kg)		Viral vector(s) injected	No. of injection tracks	No. of injection sites	Total volume injected (µl)	Experiments	Survival period
CL	Female	3.0	Left M1	AAV2-CAG-EGFP	1	2	2	Histology	1 m
				AAV5-CAG-EGFP	1	2	2	Histology	1 m
				AAV-DJ-CAG-EGFP	2	3	3	Histology	1 m
CH	Female	6.7	Left M1	AAV-DJ-CAG-H134R/tdTomato	4	8	8	oICMS/Histology	6 m
HK	Male	10.5	Left M1	AAV-DJ-CAG-H134R/tdTomato	4	8	8	oICMS/Histology	6 m
NR	Female	6.5	Left M1	AAV-DJ-CAG-H134R/EYFP	2	4	4	Histology	2 w
				AAV2-CAG-EGFP	2	4	4	Histology	2 w
				AAV5-CAG-EGFP	2	4	4	Histology	2 w
				AAV-DJ-CAG-EGFP	2	5	5	Histology	2 w
			Right M1	AAV-DJ-CAG-H134R/EYFP	4	11	11	oICMS/eICMS/Histology	2.5 m
				AAV-DJ-CAG-H134R/tdTomato	2	4	4	Histology	5 w
HJ	Female	7.5	Right M1	AAV-DJ-CAG-H134R/EYFP	5	15	15	oICMS/eICMS/Histology	5 m
PT	Female	6.1	Left M1	AAV-DJ-CAG-H134R/EYFP	5	14	14	oICMS/eICMS	*
MG	Female	5.9	Left M1	No injection	-	-	-	oICMS for controls	*

Supplementary Table 1. Summary of experiments. Survival period, period in months (m) or weeks (w) between AAV injection and sacrifice. * Monkeys PT and MG are still alive and has not been histologically verified yet. eICMS, electrical intracortical microstimulation; oICMS, optogenetic intracortical microstimulation.

Monkey	oICMS				eICMS	
	Neuronal activity (evoked sites/examined)	No. of activated neurons	Movements (evoked sites/examined)	Muscle activity (evoked sites/examined)	Movements (evoked sites/examined)	Muscle activity (evoked sites/examined)
CH	17/19	29	7/19	-	-	-
HK	44/60	88	7/60	5/10	-	-
NR	62/85	109	51/85	48/80	2/2	2/2
HJ	45/55	85	43/55	48/53	20/24	20/24
PT	22/35	48	24/25	23/24	19/38	19/38
Total	190/254	359	132/244	124/167	41/64	41/64
%	75%		54%	74%	64%	64%

Supplementary Table 2. Effects of oICMS and eICMS. Numbers of sites where oICMS evoked neuronal activity, movements, and muscle activity among numbers of sites examined, numbers of neurons activated by oICMS, and numbers of sites where eICMS evoked movements and muscle activity among numbers of sites examined, are shown. Numbers of activated neurons include multiunit activity, which was sometimes induced by oICMS. -, not examined.

Supplementary Figures

a

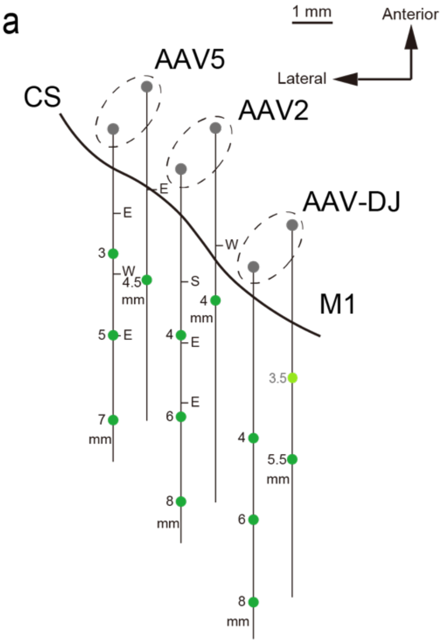

c

	Area (mm²/µl injected)	Density (cells / ROI)
AAV2	4.35	87.9
AAV5	11.05	66.8
AAV-DJ	17.65	99.7

b

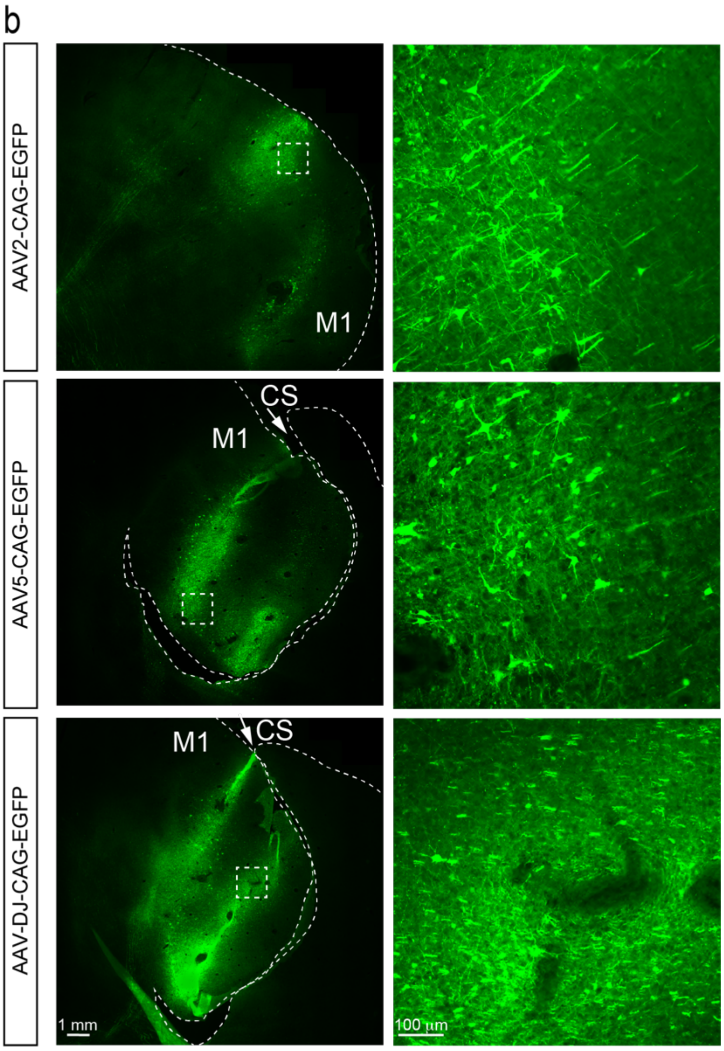

Supplementary Figure 1. Expression of EGFP transduced by AAV2, AAV5, and AAV-DJ vectors. a Injection sites in the left primary motor cortex (M1) of Monkey NR. AAV2, AAV5, and AAV-DJ vectors were injected at different depths from the cortical surface (indicated by the green circles) in the anterior bank of the central sulcus (CS). Each letter indicates a somatic body part: E, elbow; S shoulder; W wrist. Histological data at four sites of each vector injection were analyzed. The data at the light green circle by AAV-DJ injection was excluded from histological analysis. **b** Expression of EGFP mediated by AAV2, AAV5, and AAV-DJ vectors in the M1 shown in frontal sections (Monkey NR). The transduced areas and the intensity of fluorescence signals were more prominent around the injection sites of the AAV-DJ vector, compared with other serotypes. Neuropil as well as somata expressed strong fluorescence by AAV-DJ injection (*bottom right*). Areas in squares *in left* are enlarged in *right*. These experiments were repeated twice independently, with similar results obtained. **c** Areas covered by EGFP-positive cells (Monkey NR) normalized to amount of viral vector injected

(per μl) and number of EGFP-positive cells (Monkeys CL and NR) per ROI (500 μm × 500 μm). EGFP-positive cells were observed in 8, 10, and 12 slices after AAV2, AAV5, and AAV-DJ injections, respectively, in Monkey NR.

a

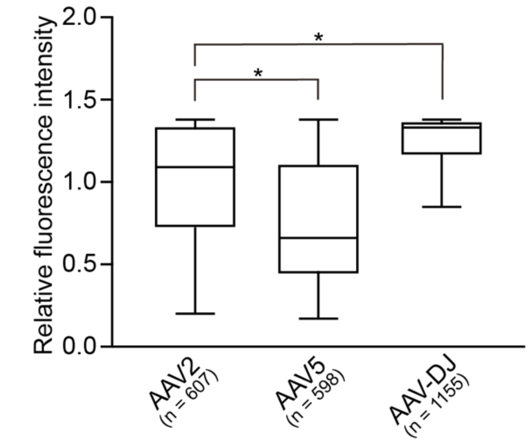

b

	NeuN / EGFP (%)	PV / EGFP (%)	GFAP / EGFP (%)
AAV2	88.3	4.6	1.8
AAV5	88.7	8.5	6.8
AAV-DJ	91.2	15.9	1.9

c

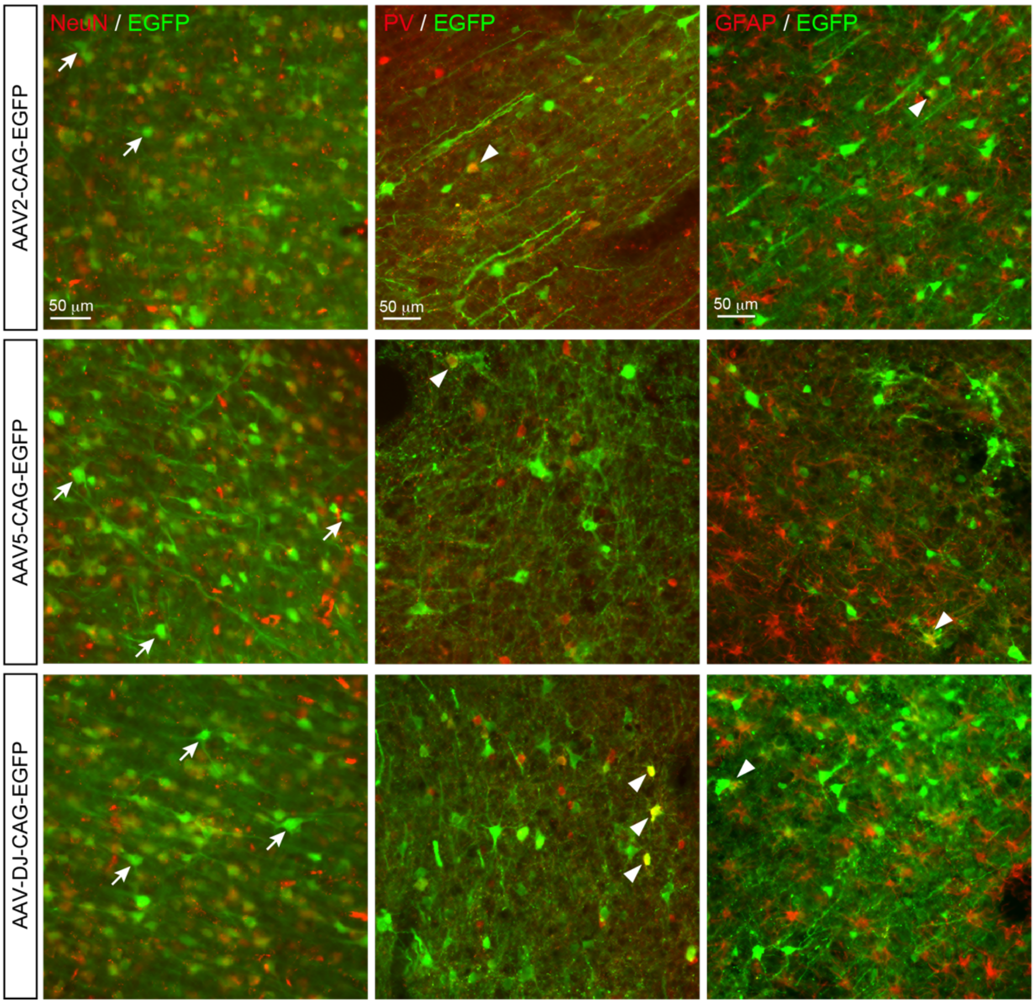

d

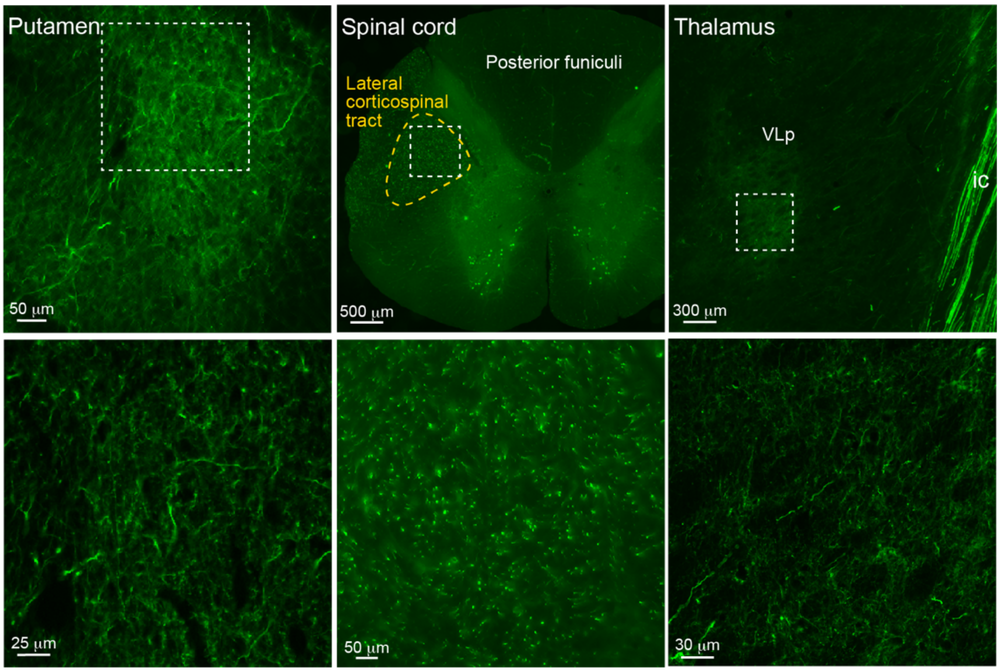

Supplementary Figure 2. Characterization of EGFP positive cells transduced by AAV2, AAV5, and AAV-DJ vectors. **a** The relative fluorescence intensity per cell by AAV2, AAV5, and AAV-DJ injection (Monkey NR) shown in box plots (center line, the median; box, upper and lower quartiles; whiskers, 1.5 × interquartile range from upper and lower quartiles). The relative fluorescence intensity was calculated as a ratio to the fluorescence intensity by AAV2 injection averaged over all cells. The number of cells used is indicated by n. * $p < 0.0001$, one-way ANOVA followed by Tukey's post hoc test. **b** Percentages of NeuN- (marker for neuron), PV- (marker for PV-positive inhibitory interneurons), and GFAP- (markers for glial cells) positive cells among all EGFP-positive cells. NeuN, PV, and GFAP were examined in Monkeys CL, NR, and NR, respectively. **c** Double staining with anti-NeuN (*left column,* Monkey CL), anti-PV (*middle column,* Monkey NR) or anti-GFAP (*right column,* Monkey NR) antibodies in combination with anti-GFP antibodies. Most EGFP-positive cells were NeuN-positive, and EGFP single-positive cells was minor (arrows). On the hand, a small population of neurons were PV/EGFP or GFAP/EGFP double-positive (arrow heads). These experiments were repeated twice independently, with similar results obtained. **d** EGFP expression in the putamen (*left*), spinal cord at the level of C7 (transverse section, *middle*), and posterior ventral lateral nucleus (VLp) of the thalamus (*right*) in Monkey CL. Areas in squares *in upper* are enlarged in *lower*. Labeled putative axon terminals in the putamen (*left*) and labeled axons in the lateral funiculus of the spinal cord, presumably the lateral corticospinal tract (circled with yellow broken lines) (*middle*) were observed. No retrogradely EGFP-labeled

cells were found in the VLp (*right*). ic, internal capsule. These experiments were repeated twice independently, with similar results obtained.

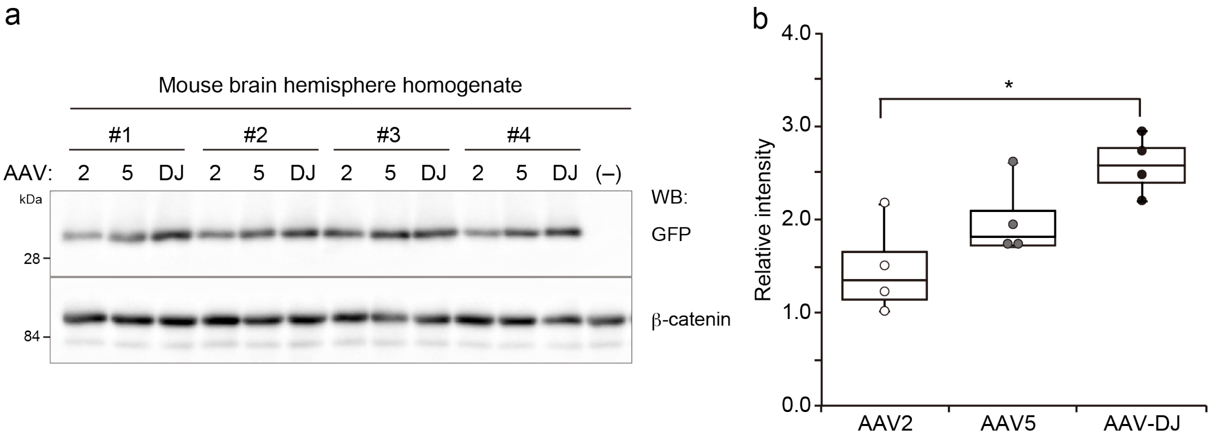

Supplementary Figure 3. Quantitative analysis of EGFP expression transduced by AAV2, AAV5, and AAV-DJ vectors. a Quantitative western blotting of brain homogenates with anti-GFP and anti-β-catenin (control) antibodies. AAV2, AAV5, or AAV-DJ vector was separately injected to both hemispheres of two mice (n = 4 for each vector). The AAV-DJ vector induced higher expression of EGFP than AAV2 and AAV5. **b** Box plots (center line, the median; box, upper and lower quartiles; whiskers, 1.5 × interquartile range from upper and lower quartiles) showing the relative intensities in AAV5 and AAV-DJ vectors as a ratio to that in experiment #1 of AAV2 (n = 4 independent samples for each vector). * $p = 0.012$, one-way ANOVA followed by Tukey's post hoc test.

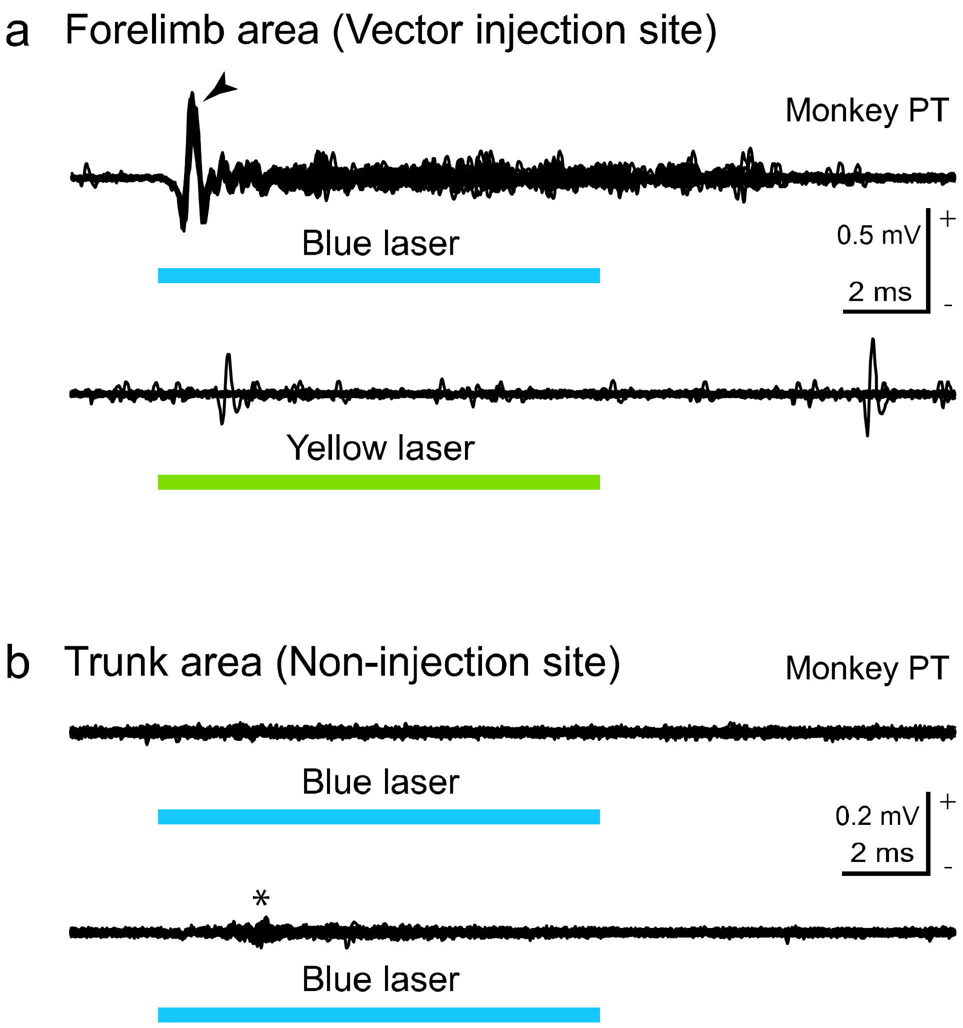

Supplementary Figure 4. Electrical potentials evoked by oICMS. a Electrical potentials recorded at a vector injection site (forelimb region of the M1). oICMS with 473 nm blue laser (15 mW strength corresponding to 1910 mW/mm^2, 10 ms pulse duration, single pulse) induced a large deflection (arrowhead in *upper trace*), probably composed of action potentials with a short and constant latency, followed by small spikes. On the other hand, oICMS with 589 nm yellow laser (15 mW, 10 ms, single pulse; 100 mW power output, COME2-589-100LS, Lucir) delivered through the same optical fiber did not induce any responses at the same recording site (*lower trace*). **b** Electrical potentials recorded at a non-injection site (trunk region of the M1). oICMS with 473 nm blue laser (15 mW, 10 ms, single pulse) induced no responses (*upper trace*) or only small fluctuations of the baseline (* in *lower trace*), probably synaptically induced by excited axon terminals of hChR2 expressing cortical neurons. These observations suggest that the large deflection observed in the upper trace in **a** is derived from neuronal activity, but not from a nonspecific photoelectric effect. Similar recordings were obtained in 13 sites in Monkey PT and 15 sites in Monkey MG.

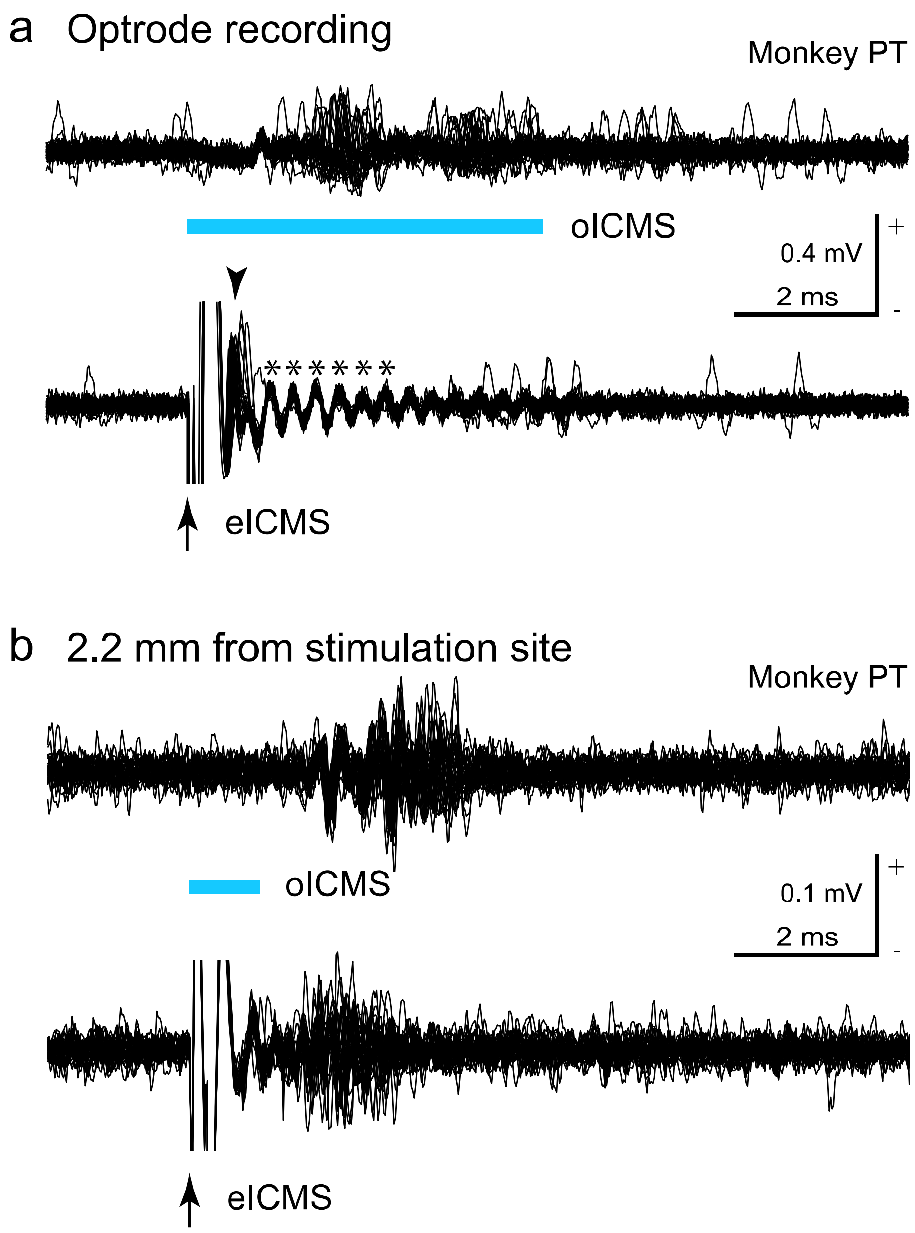

Supplementary Figure 5. Neuronal activity evoked by oICMS and eICMS. a Neuronal activity recorded with an optrode. oICMS (15 mW strength corresponding to 1910 mW/mm^2, 5 ms pulse duration, single pulse) induced small spikes (*upper trace*), while eICMS (65 µA strength, 0.2 ms pulse duration, single pulse) elicited a stimulus artifact followed by a large spike (arrowhead) and oscillatory activity (*) at the same recording site (*lower trace*). **b** Neuronal activity at 2.2 mm from oICMS/eICMS site. Another recording electrode was inserted using the second micromanipulator at a horizontal (i.e., parallel to the cortical surface) distance of 2.2 mm from the optrode. oICMS (15 mW, 1 ms, single pulse) and eICMS (65 µA , 0.2 ms, single pulse) commonly induced spikes in a similar group of neurons with larger time jitter at longer latencies than those observed in **a**, suggesting that they were induced

transsynaptically by inputs from excited neurons at the stimulation site. Similar responses were obtained in other nine cases examined, where the horizontal distances between the recording and stimulation sites were 2.2 - 3.4 mm, and both were within the forelimb region of the M1.

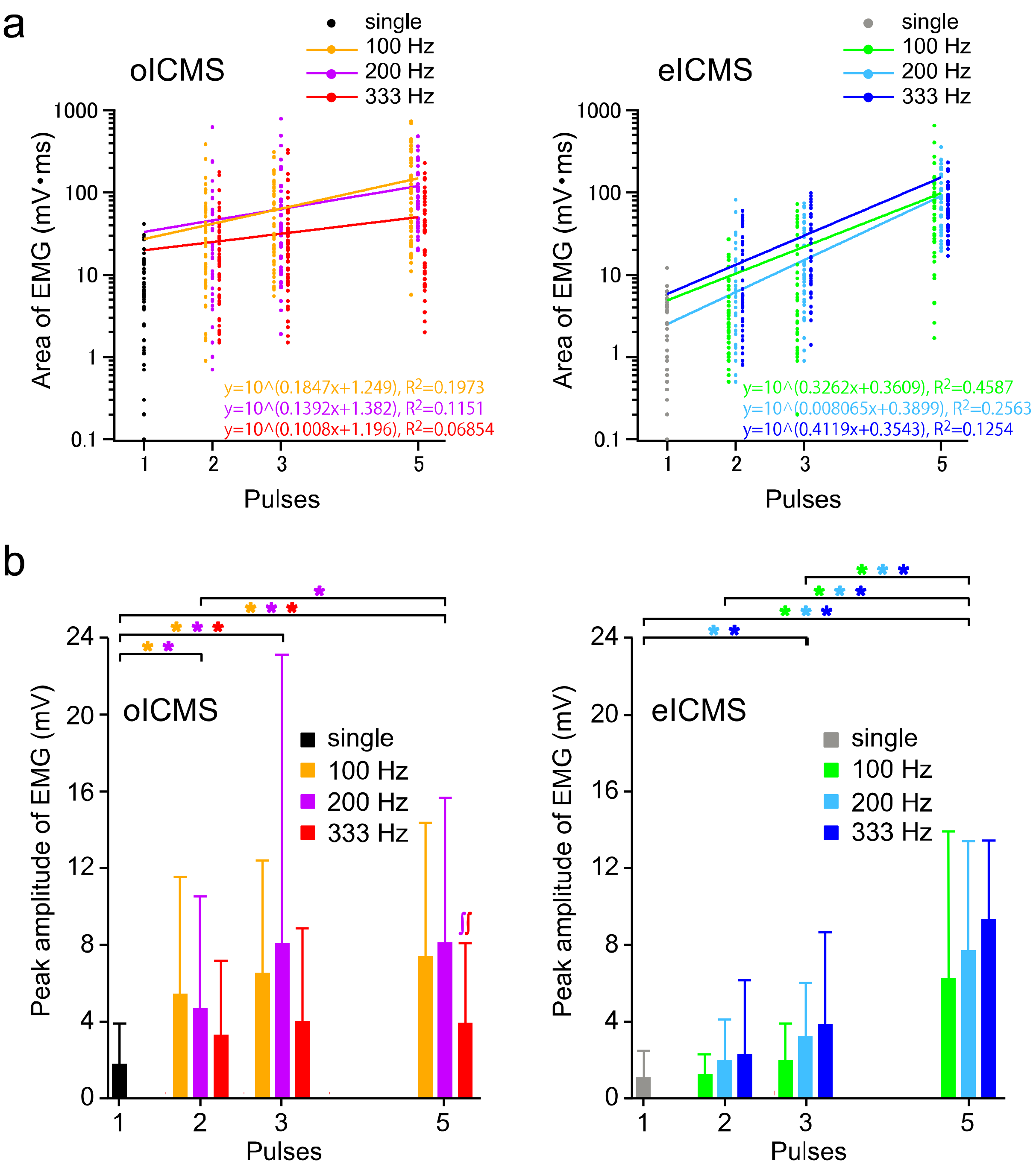

Supplementary Figure 6. The area (a) and peak amplitude (b) of EMG evoked by oICMS (*left*) and eICMS (*right*) with one, two, three, and five pulses at different frequencies.

a The logarithm of the area of EMG in each experiment is plotted against numbers of stimulus pulses in scatter graphs. Different colors indicate different frequencies (single pulse, 100, 200, and 333 Hz). Liner regression lines, regression equations, and coefficient of determination (R^2) are shown. eICMS exhibits steeper slopes of regression lines and larger R^2 than oICMS,

indicating that the area of EMG evoked by eICMS increased more rapidly than that by oICMS as the numbers of stimulus pulses increased and could be approximated by an exponential.

b The peak amplitude of EMG. The EMG data were averaged across stimulating sites (33 and 19 sites for oICMS and 19 and 20 sites for eICMS in Monkeys HJ and PT, respectively). Bar graphs represent means and SDs. * $p < 0.05$, significantly different between stimulus pulses in the frequency indicated by the color; ∫ $p < 0.05$, significantly different from each other between stimulus frequency indicated by the color (Friedman test with Dunn's post hoc test).

The peak amplitude of EMG by two, three, and five pulses of oICMS (*left*) was not different from each other ($p > 0.05$), except that the peak amplitude by two pulses was significantly smaller than that by five pulses at 200 Hz ($p < 0.0001$). One pulse of oICMS induced significantly smaller EMG than two, three, and five pulses of oICMS (two pulses, $p = 0.0003$ at 100 Hz, $p = 0.005$ at 200 Hz; three pulses, $p < 0.0001$ at 100 and 200 Hz, $p = 0.005$ at 333 Hz; five pulses, $p < 0.0001$ at 100 and 200 Hz, $p = 0.002$ at 333 Hz), except the peak amplitude by one and two pulses at 333 Hz was not different from each other ($p > 0.05$). The peak amplitude by five pulses of oICMS at 333 Hz was significantly smaller than that at 200 Hz ($p < 0.0001$).

On the other hand, the peak amplitude by one, two, and three pulses of eICMS (*right*) was all significantly smaller than that by five pulses of eICMS at 100, 200, and 333 Hz (one and two pulses, $p < 0.0001$ at 100, 200, and 333 Hz; three pulses, $p < 0.0001$ at 100 Hz, $p = 0.0007$ at 200 Hz, $p = 0.004$ at 333 Hz). The peak amplitude by one and two pulses of eICMS ($p > 0.05$), and that by two and three pulses ($p > 0.05$) was not different from each other, except that the peak amplitude by one pulse was significantly smaller than that by three pulses at 200 and 333 Hz ($p = 0.0003$ at 200 Hz, $p < 0.0001$ at 333 Hz).

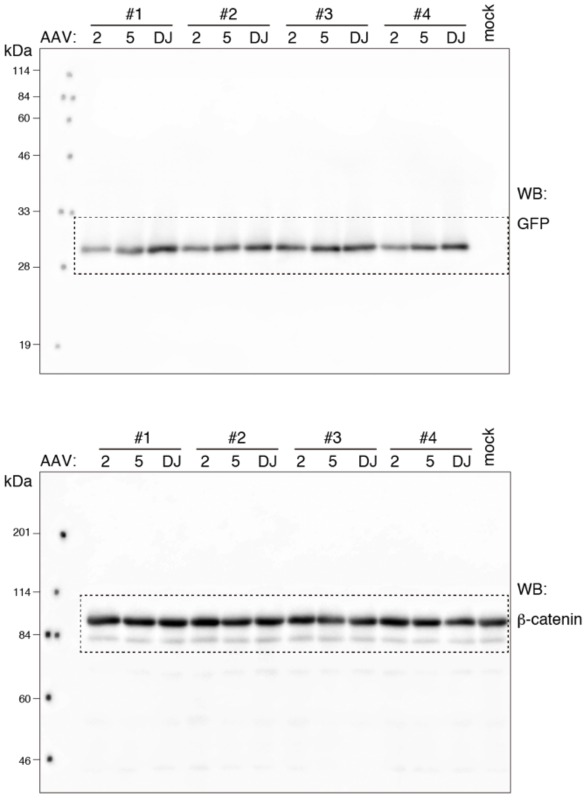

Supplementary Figure 7. Uncropped images of blots used in Supplementary Fig. 3a.
