# Supplementary material for: Forelimb movements evoked by optogenetic stimulation of the macaque motor cortex
Source: Nat Commun. 2020 Jun 26;11:3253. doi: 10.1038/s41467-020-16883-5 (PMC7319997; doi:10.1038/s41467-020-16883-5)
Supplement: Supplementary file 4 — Description of Additional Supplementary File [file 41467_2020_16883_MOESM4_ESM.docx]

Description of Additional Supplemental file

**Supplementary Movie 1**

**Forelimb movements evoked by repetitive optogenetic intracortical microstimulation (oICMS) of the macaque motor cortex**

Three typical examples of movements evoked by repetitive oICMS of the primary motor cortex: finger extension (Monkey NR; 15 mW corresponding to 1910 mW/mm^2^, 2 ms pulse duration, 250 Hz, 20 pulses), muscle twitching in the forearm (Monkey HK; 15 mW, 1 ms, 200 Hz, 20 pulses), and shoulder elevation (Monkey NR; 15 mW, 2 ms, 250 Hz, 10 pulses). The flash of LEDs in the movie indicates the timing of oICMS. The forearm and upper arm were bandaged to fix surface dish electrodes for EMG recording.
