# Supplementary material for: Forelimb movements evoked by optogenetic stimulation of the macaque motor cortex
Source: Nat Commun. 2020 Jun 26;11:3253. doi: 10.1038/s41467-020-16883-5 (PMC7319997; doi:10.1038/s41467-020-16883-5)
Supplement: Supplementary file 5 — Reporting Summary [file 41467_2020_16883_MOESM5_ESM.pdf]

nature research

Corresponding author(s): Atsushi Nambu, Hajime Mushiake

Last updated by author(s): 5/1/2020

Reporting Summary

Nature Research wishes to improve the reproducibility of the work that we publish. This form provides structure for consistency and transparency in reporting. For further information on Nature Research policies, see Authors & Referees and the Editorial Policy Checklist .

Statistics

For all statistical analyses, confirm that the following items are present in the figure legend, table legend, main text, or Methods section.

n/a	Confirmed	
[ ]	[x]	The exact sample size (n) for each experimental group/condition, given as a discrete number and unit of measurement
[ ]	[x]	A statement on whether measurements were taken from distinct samples or whether the same sample was measured repeatedly
[ ]	[x]	The statistical test(s) used AND whether they are one- or two-sided<br>*Only common tests should be described solely by name; describe more complex techniques in the Methods section.*
[x]	[ ]	A description of all covariates tested
[ ]	[x]	A description of any assumptions or corrections, such as tests of normality and adjustment for multiple comparisons
[ ]	[x]	A full description of the statistical parameters including central tendency (e.g. means) or other basic estimates (e.g. regression coefficient) AND variation (e.g. standard deviation) or associated estimates of uncertainty (e.g. confidence intervals)
[ ]	[x]	For null hypothesis testing, the test statistic (e.g. F, t, r) with confidence intervals, effect sizes, degrees of freedom and P value noted<br>*Give P values as exact values whenever suitable.*
[x]	[ ]	For Bayesian analysis, information on the choice of priors and Markov chain Monte Carlo settings
[x]	[ ]	For hierarchical and complex designs, identification of the appropriate level for tests and full reporting of outcomes
[x]	[ ]	Estimates of effect sizes (e.g. Cohen's d, Pearson's r), indicating how they were calculated

Our web collection on statistics for biologists contains articles on many of the points above.

Software and code

Policy information about availability of computer code

Data collection	LabVIEW 2013, BZ-X Viewer 1.3.0.5
Data analysis	Igor Pro 6.3, Prism 7, Prism 6, Image J 1.52k

For manuscripts utilizing custom algorithms or software that are central to the research but not yet described in published literature, software must be made available to editors/reviewers. We strongly encourage code deposition in a community repository (e.g. GitHub). See the Nature Research guidelines for submitting code & software for further information.

Data

Policy information about availability of data

All manuscripts must include a data availability statement. This statement should provide the following information, where applicable:

- Accession codes, unique identifiers, or web links for publicly available datasets
- A list of figures that have associated raw data
- A description of any restrictions on data availability

The data presented in the current manuscript are available upon request to the corresponding author (nambu@nips.ac.jp).

Field-specific reporting

Please select the one below that is the best fit for your research. If you are not sure, read the appropriate sections before making your selection.

[x] Life sciences [ ] Behavioural & social sciences [ ] Ecological, evolutionary & environmental sciences

For a reference copy of the document with all sections, see nature.com/documents/nr-reporting-summary-flat.pdf

Life sciences study design

All studies must disclose on these points even when the disclosure is negative.

Sample size	The sample size for each study was chosen based on variability estimates from other published studies.
Data exclusions	No data were excluded from the analyses.
Replication	Replication of the results was confirmed in six of six monkeys, and in two different institutes.
Randomization	Animals used in this study were randomly selected.
Blinding	The investigators were not blinded. Evoked movements were video-recorded, and evoked neuronal and muscle activity were recorded.

Reporting for specific materials, systems and methods

We require information from authors about some types of materials, experimental systems and methods used in many studies. Here, indicate whether each material, system or method listed is relevant to your study. If you are not sure if a list item applies to your research, read the appropriate section before selecting a response.

Materials & experimental systems

n/a	Involved in the study	
☐	☒	Antibodies
☐	☒	Eukaryotic cell lines
☒	☐	Palaeontology
☐	☒	Animals and other organisms
☒	☐	Human research participants
☒	☐	Clinical data

Methods

n/a	Involved in the study	
☒	☐	ChIP-seq
☒	☐	Flow cytometry
☒	☐	MRI-based neuroimaging

Antibodies

Antibodies used	Primary antibodies include: rabbit anti-GFP polyclonal (Thermo Fisher Scientific, Cat# A6455, Lot# 1736965), rabbit anti DsRed polyclonal (Takara Clontech,Cat# 632496, Lot#1612022), mouse anti-NeuN monoclonal (Merck, Cat# MAB377, Lot#LV1634819), mouse anti-GFAP monoclonal (Sigma, Cat# G3893, Lot 115K4827), mouse anti-PV monoclonal (Swant, Cat# 235, Lot# 10-11(F)), mouse b-catenin monoclonal (BD Biosciences, Cat# 610153).<br>Secondary antibodies include: Alexa488 goat anti-rabbit IgG (Thermo Fisher Scientific, Cat# A11008, Lot# 1735088), Alexa594 goat anti-rabbit IgG (Thermo Fisher Scientific, Cat# 11012, Lot# 1892265), Alexa594 goat anti-mouse IgG (Thermo Fisher Scientific, Cat# A11032, Lot# 621333).
Validation	All antibodies are in common use, and validation for each antibody were performed by suppliers. We also confirmed that controls without AAV injections were not stained expression of fluorescent proteins.

Eukaryotic cell lines

Policy information about cell lines

Cell line source(s)	293T Cell (ATCC® CRL-3216™)
Authentication	Authenticated by ATCC
Mycoplasma contamination	Cell line was not tested for mycoplasma contamination.
Commonly misidentified lines<br>(See ICLAC register)	n/a

Animals and other organisms

Policy information about studies involving animals; ARRIVE guidelines recommended for reporting animal research

Laboratory animals	Japanese monkeys (Macaca fuscata), both sex, 5-13 years old (CL, female, 5 y/o; CH, female, 11 y/o; HK, male, 13 y/o; NR, female, 9 y/o; HJ, female, 9 y/o; PT, female, 5 y/o; MG, female, 9 y/o).<br>C57BL/6J mice, male, 8 weeks old.

Wild animals	n/a
Field-collected samples	n/a
Ethics oversight	The experimental protocols were approved by the Institutional Animal Care and Use Committees and the Safety Committees for Recombinant DNA Experiments of National Institutes of Natural Sciences and Tohoku University. All experiments were conducted according to the guidelines of the National Institutes of Health Guide for the Care and Use of Laboratory Animals.

Note that full information on the approval of the study protocol must also be provided in the manuscript.
