# Supplementary material for: Forelimb movements evoked by optogenetic stimulation of the macaque motor cortex
Source: Nat Commun. 2020 Jun 26;11:3253. doi: 10.1038/s41467-020-16883-5 (PMC7319997; doi:10.1038/s41467-020-16883-5)
Supplement: Supplementary file 2 — Peer Review File [file 41467_2020_16883_MOESM2_ESM.docx]

Peer Review Information

**Journal:** Nature Communications

**Manuscript title:** Forelimb movement evoked by optogenetic stimulation of the macaque motor cortex

**Corresponding author name(s):** Prof Atsushi Nambu and Prof Hajime Mushiake

**Editorial notes:**

**Reviewer comments & decisions:**

**Reviewer comments, first version:**

Reviewer #1 (Remarks to the Author):

AAV(DJ)-CAG-ChR2 was injected into the forelimb representation of M1 of macaque monkeys. Light was delivered near the injection site via an optotrode consisting of an optical fiber, a recording electrode, and stimulating electrodes. Optical stimulation evoked trains of action potentials and movements. This result is particularly noteworthy because several previous attempts to evoke movements in primates this way failed.

Major comments

This study is largely technical. There is no discovery per se, but the behavioral results are unexpected and important. The authors are doing something right. The question is 'what?'. It is unclear which aspect of the approach used in this study overcame the obstacles that stymied previous studies. The authors propose three possibilities: the serotype of AAV used (DJ), the electrophysiological targeting of the injection to the forelimb area, and the optotrode that they used to deliver light. Any of these could be important, or some combination. Other factors may have contributed. They also used a strong ubiquitous promoter, which previous studies did not. The titer of the vector was unusually low—a fact that is unlikely to account for the result but should probably be mentioned. The study would be more useful if it shed light on which factor(s) contributed most to the behavioral result.

A rigorous quantification of the movements evoked across all of the experiments, broken down by monkey is needed.

Minor comments

Line 68–69: Data in Figures 1A & 1B are from monkey HJ, but data in Figure 1C is not. This is a bit confusing. Monkey HJ was injected with AAV-CAG-ChR2 vectors and used in electrophysiological (Figure 2B) and behavioral experiments (Figure 3A). As I understand it, the data in Figure 1C was from a monkey that was sacrificed 3 months after injection with AAV-CAG-eGFP vectors. Please indicate which monkeys were injected with which vectors, which experiments were performed in each monkey, and how long each monkey lived after the injections. Please indicate which monkey's brain sections are shown in Figure 1C. Was this monkey used in the behavioral experiments? If so, I do not understand how histological data from this monkey could have been used to select AAV-DJ for the behavioral experiments. Please clarify.

Line 71: "the four sites". Which four sites? Which vector was injected into which site? In which animal(s)?

Line 101: The text says "all four monkeys" but the figure shows data from two and the text implies that at least one monkey was used for an initial screen of serotypes (carrying CAG-eGFP, not CAG-ChR2). Again, a table would help here.

Figure 2A: The truncated action potentials and persistence of the response after the laser is turned off are striking. Have either of these phenomena been reported before? Can the authors speculate on why the responses they recorded to brief light pulses lasted so long?

Line 138: How many monkeys were used in the behavioral experiments?

Figure 4: Was the mean taken across animals? Stimulation sites? Trials? Were optical and electrical stimuli tested at the same sites? Please show data broken down by animal and document at how many stimulation sites movements were evoked.

Figure 4C: The conclusion that oICMS shows lower temporal summation than eICMS seems speculative. Even single pulses of oICMS and eICMS produced different responses, which cannot be due to differences in temporal summation. Other factors likely contribute to the difference between optical and electrical stimulation (e.g. intensity, pulse duration, population of neurons stimulated).

line 185: "monotonously" -> "monotonically"

line 216: "in another study...(ref. 15)". Reference 15 was cited in the previous sentence, so describing it as "another study" is confusing.

Line 268: typo: "The current our study".

Line 354 and 605: "jacked" -> "jacketed"

Line 363: "...head fixed in a stereotaxic frame under awake conditions..." Please provide more information about this configuration.


Reviewer #3 (Remarks to the Author):

In this manuscript, Watanabe et al. use optogenetic activation of M1 in monkeys to elicit forelimb movements. The optogenetic stimulation evokes movements that are very similar to those evoked by traditional electrical stimulation of this cortical region. The results are remarkable because other authors have previously shown that optogenetic stimulation of M1 in macaques does not result in movements, even when the optogenetic stimulation results in clear modulation of neuronal activity in these regions. The manuscript presents a side by side comparison of forelimb movements evoked by optogenetic and electrical stimulation, with simultaneous extracellular recordings in the forelimb region of M1. The comparison provides clear evidence that the optogenetic stimulation is effective to induce movements. Given that the use of optogenetics in non-human primates has been very sparse, mostly due to technical limitations, these experiments are clearly encouraging and would eventually help to move the field forward. However, the manuscript by Watanabe et al. does not provide a solid explanation of the mechanisms behind the successful optogenetic stimulation.
Even though the authors discuss that the techniques they used were instrumental to evoke movements, these techniques, at least based on the data provided, do not differ significantly from what other authors have used. The authors provide the following reasons, and I will try to explain why I do not think these are different from previous papers:
a. Use of AAV-DJ: The manuscript states that this is a very effective serotype to transduce neurons. This is not clear based on the histologic images provided. The images are only low magnification fluorescent fields. It is difficult to assess, based on these examples, what is the extent of the transduction and what types of cells were transduced. Based on these images, the extent of transduction seems similar to that reported by Diester et al 2011 (see fig 7 in that paper), but Diester et al noted that they did not observe movements in response to optical stimulation of M1, even though the proportion of neurons transduced was relatively high. In the Watanabe et al manuscript, there is no quantification of number of neurons transduced.
b. AAV vector injected in the area somatotopically identified as the forelimb region of M1: Although this is definitively a strong point of this paper, it still does not fully explain why the optogenetic stimulation was successful in this case. In Diester et al 2011 the optogenetic stimulation was not effective to evoke movements in the same location where electrical stimulations elicited movements.
c. Use of an optrode that combines an optic fiber, recording electrode and stimulation electrode: Making a probe that combines recording and optical and electrical stimulation is technically impressive. However, it is not clear why the probe itself would help to achieve optogenetic-evoked movement. The basic design (flat end fiber, glued to electrode) is similar to most other optrodes described in non-human primate optogenetic papers (see for example Galvan et al 2012, Stauffer et al 2016). One important difference, however, is that the distance between the tip of the fiber and the tip of the electrode is larger than in previous publications. I wonder if this might have contributed to evoke movement (see below for more on this point).

So, to summarize, the 3 reasons outlined above do not seem to be enough to explain the results of these paper. Furthermore, in the introduction the authors speculate that the reasons why previous papers have failed to induce movements with optogenetic stimulation cold be due to insufficient opsin expression or poor light penetration. Again, the data in the manuscript does not seem to indicate that the techniques used overcame these issues.
it remains unclear how the techniques described in this paper were successful to induce optogenetically-evoked forelimb movement. So, in my point of view, the main question of why these experiments achieved optogenetic-evoked movement, when previous papers could not show this, remains unanswered. This is the major problem of this paper, and although the data is very exciting, the authors need to address this issue.
I will now list some other comments to the paper, and some suggestions about possible mechanisms that the authors may want to consider that could be contributing to the results.

1. The histology, as mentioned above, is extremely limited. If the authors think that the extent of virus transduction by AAV-DJ is key to their success, a detailed description of the histology would be very important. The results should include a quantification of the extent of the areas covered by the transduction, type of cells transduced (glia, projection neurons, interneurons), quantification of neurons expressing the opsin, description of anterograde and retrograde transport of the opsins. Higher magnification images should be provided.
2. Why was a non-specific promoter (CAG) used for these studies? It is likely that there is at least some degree of non-neuronal transduction (again, a more detailed description of the histological results would be useful). It may be possible that opsin expression in glia (which has been avoided in previous publications by using neuronal promoters) could be important to achieve optogenetic-mediated movement in monkeys.
3. The comparisons of the results obtained with the three different AAVs (AAV2, AAV5 and AAV-DJ) needs more work. It is not clear what is the extent of the transduction achieved by each serotype. Was there overlap of the expression? The injections were only 1 mm apart, and some overlap would be expected.
4. The decision to use AAV-DJ seems to be based only on qualitative comparisons of the extent of the fluorescence obtained in the 3 cases. A more rigorous comparison would be appreciated to help understand why this serotype is superior to the others.
5. Light intensity should be provided as mW/mm2, and these should be compared with what was used in previous papers. For example, the results indicate that less than 1 mW of light was sufficient to evoke spikes. With the 100 um core fiber, 1 mW correspond roughly to 127 mW/mm2, in the range of that used in previous papers for M1 stimulation (i. e., Diester et al 2011; Galvan et al 2016)
6. I am very intrigued about the possibility that the distance between the tip of the recording electrode and the optic fiber could be a determinant factor. If, during the recording experiments, the electrode is recording neurons in deep cortical layers, the fiber is positioned 800-100 um dorsally, likely illuminating superficial and deep cortical layers. In previous papers, like Diester et al 2011, the electrode was 200-400 um from the fiber tip, and thus, if the electrode is in deep layers, the light will target only these layers, and perhaps the white matter ventral to it. This possibility could be explored to help explain the results in this manuscript.
7. The data in figure 2 are very difficult to interpret. Are traces in panels a through c from the same neuron? Without spike sorting, it is not clear how many units were close to the recording site, and which units were modulated by the optogenetic stimulation. Most importantly: do the authors think that the depolarizations, multi-unit excitations and complex responses were related to the evoked movement? To answer this question, it may be useful to see recordings obtained during the electrical stimulation. Obviously, the artifacts of the electrical stimulation will obscure some of the recordings, but the artifacts can be digitally removed offline, allowing a comparison of the neuronal responses obtained during optogenetic and electrical stimulation
8. Please provide details regarding how many neurons were recorded during optogenetic stimulation (and how many neurons were obtained from each monkey), and the proportion of neurons that were modulated by the stimulation. It would be useful to compare these proportions to those reported in other studies doing optogenetic stimulation of M1 in monkeys.
9. Why use such very high frequencies (100 to 333 Hz) to drive ChR2? It is known that this opsin cannot follow such high frequencies (see Mattis et al 2012, Gunaydin et al. 2010). Were the neurons in cortex able to follow these frequencies? Were lower frequencies tested to see if those also induce movements?
10. Light intensities used to evoke movements cover a wide range (0.4 to 15 mW). Were all these equally effective, or was there an optimal intensity?
11. The side by side comparison of electrical and optogenetic stimulation is one of the major strengths of this manuscript. It would be helpful to interpret the results to include some estimates of the extent of the area stimulated by one or the other method.
12. The electrical stimulation and optogenetic stimulation were delivered in the same location. Is it possible that the electrical stimulation ‘primed’ or ‘kindled’ the optogenetic stimulation? Were there controls in which only one the optogenetic stimulation was conducted?
13. The simultaneous recordings in M1 with EMG recordings are a great set of experiments. They rise a lot of important questions. It seems that the neuronal activity in M1 shown in figure 3a and 3b is, in fact, reduced during optogenetic stimulation (at least for the large unit). Is this so? Can a quantification of neuronal activity be provided to help assess this issue?
14. Along the same line, the results shown that the changes in the EMG traces evoked by 333 Hz is smaller than changes evoked by 100 and 200 Hz during optogenetic stimulation. This could be due to the inability of Chr2 to follow high frequencies.
15. Quantification of EMG responses should be better explained. For example in page 11, line 156, what does n=46 refers to? Same for line 164.
16. I recommend to plot the data in figure 4c and 4d as a bar graph instead of line-symbol graph. Also, I suggest to use SD instead of SEs in this graphs.
17. In Discussion, it is stated that optogenetic responses were observed in layer 5. But, since the expression of the opsins seems to expand many layers, it is possible that the movements evoked by optogenetic stimulation were result of activation of neurons across cortical layers.
18. Please include a table, or a description, of which AAV serotypes were injected in which monkeys, and which experiments were done in each (how many neurons obtained, how many EMG recordings made…).
19. In Methods, there is no description of the statistical analysis used.

Minor comments:
• Page 9, line 128: ‘53/69 recording sites’. This data should be expressed as number of neurons recorded (multiple unit recordings could be spike sorted to obtain number of neurons)
• Not very clear (at least to me) what is meant by ‘monotonously’ in page 12 line 186. Can other term be used to describe this response?
• Are examples in figure 2 from the same neuron?
• In figures 2 and 3: In which layer of cortex were the neurons recorded?
• Discussion: The first paragraph is not really needed. It is just a recap of what optogenetic techniques are, and there are plenty of reviews that provide this information. I suggest to remove.
• Page 14, lines 219: Mention of the use of NpHR to modulate neurons in M1 of monkeys. A reference by Kinoshita et al 2012 is cited. The work by Diester et al 2011 should also be cited here.
• Page 16, line 245 “….AAV-DJ showed the most potent gene transfer”. As mentioned above, this very strong statement should be backed up by appropriate quantification.
• Looking at figure 1, it seems that the tdTomato-containing vector was ‘less effective’ than the others. Again, a quantification could help assess if this is the case.
• In Methods, it is stated that there was a 3 week waiting period between injecitons and the start of the optogenetic experiments. Perhaps I missed it, but I did not see the waiting period for the histology experiments to compare the various AAV serotypes (shown in fig 1)
• Stat symbols in figure 4c are unclear. The symbols do not seem to correspond with the legend. Please check.
• If needed, were corrections for multiple comparisons done for the stats done in figure 4c?
• Was the histological examination conduced in all monkeys? Please clarify


Reviewer #4 (Remarks to the Author):

Watanabe et al. show that forelimb movements in monkeys can be evoked by optogenetic stimulation of motor cortex. Movements were similar to conventional electrical microstimualtion, except that there was slightly less non-linear summation. On one hand, these results are to be expected – optogenetics is widely used to activate neural circuits in rodents so it should not come as a surprise that activation of motor cortex, which projects directly to the spinal cord, should elicit movement. On the other hand, demonstrations of optogenetics in non-human primates are still relatively rare, with very few reports of behavioural effects, likely due to the larger brain size and the need to optimise viral delivery methods. Indeed, the difficulties of evoking motor potentials through optogenetics has been something of a barrier to adoption of optogenetics by the NHP neuroscience community, so it is good to see this problem resolved. Therefore demonstrating successful application of the technique is an important contribution. However, it is a shame that there is little by way of new scientific insight contained within this paper, for example using the cell-specificity enabled by optogenetics to examine more about how motor cortical circuitry is engaged by oICMS and eICMS. In addition, there is little attempt to characterize stimulation effects, e.g. what area of cortex is activated, how many cells (as was undertaken for eICMS by studies such as Stoney et al (1968)).
Therefore, in my view the manuscript should be judged as a methods paper, and in this regard there are some important areas in which it falls short of the level of detail which would aid adoption by the community.

Major comments:
Choice of virus: An important part of getting optogenetics to work in NHPs is the choice of viral vector. Here the authors compare three AAV serotypes, in the end suggesting that AAV-DJ is best. This is extremely valuable data but no quantification is provided, only some example images. Quantification of the area of infected cortex, proportion of cells infected and what type of cells should be included. Although using a ubiquitous promotor, there may be differences in infection of different cell types - this can be looked at in histology data. In addition, it would be useful to know if corticospinal tract and corticomotoneuronal cells are infected - this could be observed in spinal cord histology.
Optrode: The authors describe a new optrode for delivering light to the tissue, but there is no attempt to compare with existing commercial designs, or provide motivation for the particular design choices made for this study. In addition, optical power is given in mW, measured 5mm from the optrode tip. However, the separation between optrode tip and recording site is about 1mm. The authors should provide details on light intensity measured as power per unit area at the electrode tip - this could then be related to thresholds for opsin activation. This would require some consideration of the light spread from the fibre, but would allow easier comparison with other papers in the literature.
The truncated spikes occurring after stimulation should be better documented. These bursts appear unusual (e.g. compare with Diester et al. Nat Neurosci 2011 where spikes are elicited only during stimulation) and look a bit like injury potentials – is there a chance that the neuron could be unhealthy and/or damaged by the optrode? A figure with overlaid spikes, to convince readers that they are actually from the same neuron would help. For how long are they truncated? Is the firing pattern of these neurons normal when not stimulating, or do these cells generally fire in a burst manner (which could be an indication of injury).

Minor comments:
Line 117: Could the deflection observed when an optogenetic stimulation is delivered be a photoelectric effect? A common control is to use a light with a different wavelength. Instead it is stated that the deflection was seen “observed only at the areas where ChR2 was expressed” but this important point is not shown or quantified in the paper.
Line 153: the authors should specify if the EMG responses were recorded whilst the primate was performing any specific behaviour.
Line 185: oICMS shows lower temporal summation than eICMS. This refers to figure 4. There may be another explanation in that single pulse oICMS is produces a response that is three time higher than single pulse eICMS. Therefore it is possible that the response to extra stimulation pulses is saturated.
Methods: Please state which muscles were recorded from.

**Author rebuttal, first version:**

**Reviewer #1 (Remarks to the Author):**
AAV(DJ)-CAG-ChR2 was injected into the forelimb representation of M1 of macaque monkeys. Light was delivered near the injection site via an optotrode consisting of an optical fiber, a recording electrode, and stimulating electrodes. Optical stimulation evoked trains of action potentials and movements. This result is particularly noteworthy because several previous attempts to evoke movements in primates this way failed.

**Major comments**

This study is largely technical. There is no discovery per se, but the behavioral results are unexpected and important. The authors are doing something right. The question is 'what?'. It is unclear which aspect of the approach used in this study overcame the obstacles that stymied previous studies. The authors propose three possibilities: the serotype of AAV used (DJ), the electrophysiological targeting of the injection to the forelimb area, and the optotrode that they used to deliver light. Any of these could be important, or some combination. Other factors may have contributed. They also used a strong ubiquitous promoter, which previous studies did not. The titer of the vector was unusually low—a fact that is unlikely to account for the result but should probably be mentioned. The study would be more useful if it shed light on which factor(s) contributed most to the behavioral result.

Thank you for your suggestions.

We pointed out four factors (page 16-17).

The key to our success in inducing obvious movements by optogenetics in macaque monkeys was a winning combination of the following: (1) A highly effective AAV serotype DJ and a strong ubiquitous promoter CAG enabled potent gene transfer of ChR2 into neurons, (2) The AAV vector was injected into somatotopically identified regions of the M1, (3) Our ingenious optrode could identify layer 5 cortical neurons expressing ChR2 and illuminate both superficial and deep cortical layers expressing ChR2 by the optical fiber, and (4) Higher intensity of laser (51-1910 mW/mm^2^) was used for illumination than in previous studies (3-1600 mW/mm^2^).

We also added Supplementary Figures 1, 2 and 3 supporting effectiveness of AAV-DJ vector.

We have corrected titer of the vector (from ml to µl).

A rigorous quantification of the movements evoked across all of the experiments, broken down by monkey is needed.

We have added Supplementary Table 2.

**Minor comments**

Line 68–69: Data in Figures 1A & 1B are from monkey HJ, but data in Figure 1C is not. This is a bit confusing. Monkey HJ was injected with AAV-CAG-ChR2 vectors and used in electrophysiological (Figure 2B) and behavioral experiments (Figure 3A). As I understand it, the data in Figure 1C was from a monkey that was sacrificed 3 months after injection with AAV-CAG-eGFP vectors. Please indicate which monkeys were injected with which vectors, which experiments were performed in each monkey, and how long each monkey lived after the injections. Please indicate which monkey's brain sections are shown in Figure 1C. Was this monkey used in the behavioral experiments? If so, I do not understand how histological data from this monkey could have been used to select AAV-DJ for the behavioral experiments. Please clarify.

We had evaluated viral vectors using Monkey CL (Supplementary Figs. 1 and 2), which was not included in the previous version of the manuscript, because we did not perform electrophysiological experiment in this monkey. Now, we added data of Monkey CL to the revised manuscript and discussed the evaluation.

We have added Supplementary Table 1. We also added the name of the monkey to the figure legends.

Line 71: "the four sites". Which four sites? Which vector was injected into which site? In which animal(s)?

We have added Supplementary Table 1. We injected into 4 sites as indicated in Supplementary Fig. 1a.

Line 101: The text says "all four monkeys" but the figure shows data from two and the text implies that at least one monkey was used for an initial screen of serotypes (carrying CAG-eGFP, not CAG-ChR2). Again, a table would help here.

We have added Supplementary Table 1.

Figure 2A: The truncated action potentials and persistence of the response after the laser is turned off are striking. Have either of these phenomena been reported before? Can the authors speculate on why the responses they recorded to brief light pulses lasted so long?

We added the following explanation (page 10).

This phenomenon is interpreted that high-intensity oICMS induces large depolarization in cortical neurons, which lasts long after the cession of laser illumination (up to 10 ms), and truncates the shapes of action potentials because of depolarization block. These complex responses evoked by oICMS were also observed in eICMS (Supplementary Fig. 5a).

We also cited the reference 21: Herman et al. *Elife* **3**, e01481 (2014). They showed depolarization block by optogenetic stimulation.

Line 138: How many monkeys were used in the behavioral experiments?

To analyze EMG systematically, we added another monkey (Monkey PT). Thus, we totally used five monkeys (see Supplementary Table 1).

Figure 4: Was the mean taken across animals? Stimulation sites? Trials? Were optical and electrical stimuli tested at the same sites? Please show data broken down by animal and document at how many stimulation sites movements were evoked.

We added the following sentence to the figure legend of Fig. 4 (page 39-40).

Both oICMS and eICMS were applied, and the EMG data were averaged across stimulating sites (33 and 19 sites for oICMS and 19 and 20 sites for eICMS in Monkeys HJ and PT, respectively).

We have also added Supplementary Table 2.

We applied oICMS and eICMS in the same site in Fig. 4a, but not always in Fig. 4b.

Figure 4C: The conclusion that oICMS shows lower temporal summation than eICMS seems speculative. Even single pulses of oICMS and eICMS produced different responses, which cannot be due to differences in temporal summation. Other factors likely contribute to the difference between optical and electrical stimulation (e.g. intensity, pulse duration, population of neurons stimulated).

We agree with the reviewer’s comment.

We deleted the description on temporal summation. Instead, we have added following description (page 14).

These results suggest that oICMS induces muscle activity more effectively than eICMS at fewer pulses.

We added the following sentences to the Discussion section (page 19-20).

These results suggest that oICMS induces body movements more effectively than eICMS. One reason is that oICMS illuminates neural tissue along the long axis and may effectively activate the cortical efferent zone. On the other hand, eICMS may activate neurons inside a sphere around the electrode tip, the size of which increases with increasing current.

line 185: "monotonously" -> "monotonically"

We deleted this sentence to avoid confusion.

line 216: "in another study...(ref. 15)". Reference 15 was cited in the previous sentence, so describing it as "another study" is confusing.

We quitted citing this reference in this sentence (page 16).

Line 268: typo: "The current our study".

We corrected as suggested (page 20).

Line 354 and 605: "jacked" -> "jacketed"

We corrected as suggested (page 25, 37).

Line 363: "...head fixed in a stereotaxic frame under awake conditions..." Please provide more information about this configuration.

We added the following description (page 26).

Each monkey was seated in a monkey chair under awake conditions. Two stainless rods were inserted into PEEK pipes over its head and were fixed in a stereotaxic frame without any pain as described previously.

We also cited References 43 and 44.


**Reviewer #3 (Remarks to the Author):**

In this manuscript, Watanabe et al. use optogenetic activation of M1 in monkeys to elicit forelimb movements. The optogenetic stimulation evokes movements that are very similar to those evoked by traditional electrical stimulation of this cortical region. The results are remarkable because other authors have previously shown that optogenetic stimulation of M1 in macaques does not result in movements, even when the optogenetic stimulation results in clear modulation of neuronal activity in these regions. The manuscript presents a side by side comparison of forelimb movements evoked by optogenetic and electrical stimulation, with simultaneous extracellular recordings in the forelimb region of M1. The comparison provides clear evidence that the optogenetic stimulation is effective to induce movements. Given that the use of optogenetics in non-human primates has been very sparse, mostly due to technical limitations, these experiments are clearly encouraging and would eventually help
to move the field forward. However, the manuscript by Watanabe et al. does not provide a solid explanation of the mechanisms behind the successful optogenetic stimulation.
Even though the authors discuss that the techniques they used were instrumental to evoke movements, these techniques, at least based on the data provided, do not differ significantly from what other authors have used. The authors provide the following reasons, and I will try to explain why I do not think these are different from previous papers:

a. Use of AAV-DJ: The manuscript states that this is a very effective serotype to transduce neurons. This is not clear based on the histologic images provided. The images are only low magnification fluorescent fields. It is difficult to assess, based on these examples, what is the extent of the transduction and what types of cells were transduced. Based on these images, the extent of transduction seems similar to that reported by Diester et al 2011 (see fig 7 in that paper), but Diester et al noted that they did not observe movements in response to optical stimulation of M1, even though the proportion of neurons transduced was relatively high. In the Watanabe et al manuscript, there is no quantification of number of neurons transduced.

We added following description (Page 6-7).

The fluorescence signals were most conspicuous around the injection sites of the AAV-DJ vector (Supplementary Fig. 1b). The areas and number of cells transduced by the AAV-DJ vector were larger than those transduced by AAV2 and AAV5 (Supplementary Fig. 1c). The intensity of fluorescence signals at the single-neuron level by the AAV-DJ vector was higher than that by AAV2 and AAV5 (Supplementary Fig. 2a). Observing expression of NeuN, parvalbumin (PV), and glial fibrillary acidic protein (GFAP) among EGFP positive cells (Supplementary Fig. 2b, c) showed that the major cell type expressing EGFP was NeuN-positive neurons, and that glial cells expressing EGFP were a small population (Supplementary Fig. 2b). The AAV-DJ vector induced EGFP expression at terminals in the putamen and at axons presumably in the lateral corticospinal tract of the spinal cord, but no retrogradely labeled cells were found in the thalamus (Supplementary Fig. 2d). In addition, the EGFP protein level induced by the AAV-DJ vector in the mouse brain was higher than that induced by AAV2 and AAV5 (Supplementary Fig. 3).

We added Supplementary Figs. 1, 2, and 3.

b. AAV vector injected in the area somatotopically identified as the forelimb region of M1: Although this is definitively a strong point of this paper, it still does not fully explain why the optogenetic stimulation was successful in this case. In Diester et al 2011 the optogenetic stimulation was not effective to evoke movements in the same location where electrical stimulations elicited movements.

We added the following explanation (page 16-17).

The key to our success in inducing obvious movements by optogenetics in macaque monkeys was a winning combination of the following: (1) A highly effective AAV serotype DJ and a strong ubiquitous promoter CAG enabled potent gene transfer of ChR2 into neurons, (2) The AAV vector was injected into somatotopically identified regions of the M1, (3) Our ingenious optrode could identify layer 5 cortical neurons expressing ChR2 and illuminate both superficial and deep cortical layers expressing ChR2 by the optical fiber, and (4) Higher intensity of laser (51-1910 mW/mm^2^) was used for illumination than in previous studies (3-1600 mW/mm^2^).

c. Use of an optrode that combines an optic fiber, recording electrode and stimulation electrode: Making a probe that combines recording and optical and electrical stimulation is technically impressive. However, it is not clear why the probe itself would help to achieve optogenetic-evoked movement. The basic design (flat end fiber, glued to electrode) is similar to most other optrodes described in non-human primate optogenetic papers (see for example Galvan et al 2012, Stauffer et al 2016). One important difference, however, is that the distance between the tip of the fiber and the tip of the electrode is larger than in previous publications. I wonder if this might have contributed to evoke movement (see below for more on this point).

Thank you for your suggestion. We added the following sentence (page 16-17).

Our ingenious optrode could identify layer 5 cortical neurons expressing ChR2 and illuminate both superficial and deep cortical layers expressing ChR2 by the optical fiber

So, to summarize, the 3 reasons outlined above do not seem to be enough to explain the results of these paper. Furthermore, in the introduction the authors speculate that the reasons why previous papers have failed to induce movements with optogenetic stimulation cold be due to insufficient opsin expression or poor light penetration. Again, the data in the manuscript does not seem to indicate that the techniques used overcame these issues.
it remains unclear how the techniques described in this paper were successful to induce optogenetically-evoked forelimb movement. So, in my point of view, the main question of why these experiments achieved optogenetic-evoked movement, when previous papers could not show this, remains unanswered. This is the major problem of this paper, and although the data is very exciting, the authors need to address this issue.

We added the following explanation (page 16-17).

The key to our success in inducing obvious movements by optogenetics in macaque monkeys was a winning combination of the following: (1) A highly effective AAV serotype DJ and a strong ubiquitous promoter CAG enabled potent gene transfer of ChR2 into neurons, (2) The AAV vector was injected into somatotopically identified regions of the M1, (3) Our ingenious optrode could identify layer 5 cortical neurons expressing ChR2 and illuminate both superficial and deep cortical layers expressing ChR2 by the optical fiber, and (4) Higher intensity of laser (51-1910 mW/mm^2^) was used for illumination than in previous studies (3-1600 mW/mm^2^).

We added experiments (Supplementary Fig. 1, 2, 3) supporting above hypothesis (1) and added the following description (page 6-7).

The fluorescence signals were most conspicuous around the injection sites of the AAV-DJ vector (Supplementary Fig. 1b). The areas and number of cells transduced by the AAV-DJ vector were larger than those transduced by AAV2 and AAV5 (Supplementary Fig. 1c). The intensity of fluorescence signals at the single-neuron level by the AAV-DJ vector was higher than that by AAV2 and AAV5 (Supplementary Fig. 2a). Observing expression of NeuN, parvalbumin (PV), and glial fibrillary acidic protein (GFAP) among EGFP positive cells (Supplementary Fig. 2b, c) showed that the major cell type expressing EGFP was NeuN-positive neurons, and that glial cells expressing EGFP were a small population (Supplementary Fig. 2b). The AAV-DJ vector induced EGFP expression at terminals in the putamen and at axons presumably in the lateral corticospinal tract of the spinal cord, but no retrogradely labeled cells were found in the thalamus (Supplementary Fig. 2d). In addition, the EGFP protein level induced by the AAV-DJ vector in the mouse brain was higher than that induced by AAV2 and AAV5 (Supplementary Fig. 3).

Concerning the intensity of laser light, we added the following description to the Discussion section (page 18-19).

In the present study, we used high intensity of laser (51-1910 mW/mm^2^) compared to previous studies. Previous studies targeting M1 cortical neurons expressing ChR2 used weaker laser (3-255 mW/mm^2^)^12,13^ and could not affect behaviors. On the other hand, other studies used higher-intensity laser to inhibit Archaerhodopsin-T expressing superior collicular neurons (650-1600 mW/mm^2^) or to excite ChR2-expressing terminals in the superior colliculus (<1000 mW/mm^2^) and successfully affected eye movements. Thus, we decided to use higher-intensity laser in this study.

I will now list some other comments to the paper, and some suggestions about possible mechanisms that the authors may want to consider that could be contributing to the results.

1. The histology, as mentioned above, is extremely limited. If the authors think that the extent of virus transduction by AAV-DJ is key to their success, a detailed description of the histology would be very important. The results should include a quantification of the extent of the areas covered by the transduction, type of cells transduced (glia, projection neurons, interneurons), quantification of neurons expressing the opsin, description of anterograde and retrograde transport of the opsins. Higher magnification images should be provided.

We added experiments (Supplementary Fig. 1, 2, 3) supporting above hypothesis (1) and added the following description (page 6-7).

The fluorescence signals were most conspicuous around the injection sites of the AAV-DJ vector (Supplementary Fig. 1b). The areas and number of cells transduced by the AAV-DJ vector were larger than those transduced by AAV2 and AAV5 (Supplementary Fig. 1c). The intensity of fluorescence signals at the single-neuron level by the AAV-DJ vector was higher than that by AAV2 and AAV5 (Supplementary Fig. 2a). Observing expression of NeuN, parvalbumin (PV), and glial fibrillary acidic protein (GFAP) among EGFP positive cells (Supplementary Fig. 2b, c) showed that the major cell type expressing EGFP was NeuN-positive neurons, and that glial cells expressing EGFP were a small population (Supplementary Fig. 2b). The AAV-DJ vector induced EGFP expression at terminals in the putamen and at axons presumably in the lateral corticospinal tract of the spinal cord, but no retrogradely labeled cells were found in the thalamus (Supplementary Fig. 2d). In addition, the EGFP protein level induced by the AAV-DJ vector in the mouse brain was higher than that induced by AAV2 and AAV5 (Supplementary Fig. 3).

2. Why was a non-specific promoter (CAG) used for these studies? It is likely that there is at least some degree of non-neuronal transduction (again, a more detailed description of the histological results would be useful). It may be possible that opsin expression in glia (which has been avoided in previous publications by using neuronal promoters) could be important to achieve optogenetic-mediated movement in monkeys.

We used a strong ubiquitous promoter CAG to induce higher expression of EGFP or ChR2. We added following description (page 7).

Therefore, we chose the AAV-DJ vector and generated a vector containing channelrhodopsin-2 (ChR2) with a point mutation [hChR2(H134R)] and a strong ubiquitous promoter CAG.

EGFP expression in glial cells was not high. We described as follows (page 6-7). Please see also Supplementary Fig. 2.

Observing expression of NeuN, parvalbumin (PV), and glial fibrillary acidic protein (GFAP) among EGFP positive cells (Supplementary Fig. 2b, c) showed that the major cell type expressing EGFP was NeuN-positive neurons, and that glial cells expressing EGFP were a small population (Supplementary Fig. 2b).

3. The comparisons of the results obtained with the three different AAVs (AAV2, AAV5 and AAV-DJ) needs more work. It is not clear what is the extent of the transduction achieved by each serotype. Was there overlap of the expression? The injections were only 1 mm apart, and some overlap would be expected.

Each injection site was separated at least 2 mm (Supplementary Fig. 1a), and there was no overlap.

We compared three AAVs and added the following description (page 6-7).

Observing expression of NeuN, parvalbumin (PV), and glial fibrillary acidic protein (GFAP) among EGFP positive cells (Supplementary Fig. 2b, c) showed that the major cell type expressing EGFP was NeuN-positive neurons, and that glial cells expressing EGFP were a small population (Supplementary Fig. 2b). The AAV-DJ vector induced EGFP expression at terminals in the putamen and at axons presumably in the lateral corticospinal tract of the spinal cord, but no retrogradely labeled cells were found in the thalamus (Supplementary Fig. 2d). In addition, the EGFP protein level induced by the AAV-DJ vector in the mouse brain was higher than that induced by AAV2 and AAV5 (Supplementary Fig. 3).

4. The decision to use AAV-DJ seems to be based only on qualitative comparisons of the extent of the fluorescence obtained in the 3 cases. A more rigorous comparison would be appreciated to help understand why this serotype is superior to the others.

We analyzed also quantitatively. Please see Supplementary Figs. 1c, 2ab, 3

5. Light intensity should be provided as mW/mm2, and these should be compared with what was used in previous papers. For example, the results indicate that less than 1 mW of light was sufficient to evoke spikes. With the 100 um core fiber, 1 mW correspond roughly to 127 mW/mm2, in the range of that used in previous papers for M1 stimulation (i. e., Diester et al 2011; Galvan et al 2016)

We added the light intensity expressed in mW/mm2 to the main text and figure legends.

6. I am very intrigued about the possibility that the distance between the tip of the recording electrode and the optic fiber could be a determinant factor. If, during the recording experiments, the electrode is recording neurons in deep cortical layers, the fiber is positioned 800-100 um dorsally, likely illuminating superficial and deep cortical layers. In previous papers, like Diester et al 2011, the electrode was 200-400 um from the fiber tip, and thus, if the electrode is in deep layers, the light will target only these layers, and perhaps the white matter ventral to it. This possibility could be explored to help explain the results in this manuscript.

Thank you for your suggestion. It is probable. We added the following sentence (page 16-17).

Our ingenious optrode could identify layer 5 cortical neurons expressing ChR2 and illuminate both superficial and deep cortical layers expressing ChR2 by the optical fiber.

7. The data in figure 2 are very difficult to interpret. Are traces in panels a through c from the same neuron? Without spike sorting, it is not clear how many units were close to the recording site, and which units were modulated by the optogenetic stimulation. Most importantly: do the authors think that the depolarizations, multi-unit excitations and complex responses were related to the evoked movement? To answer this question, it may be useful to see recordings obtained during the electrical stimulation. Obviously, the artifacts of the electrical stimulation will obscure some of the recordings, but the artifacts can be digitally removed offline, allowing a comparison of the neuronal responses obtained during optogenetic and electrical stimulation

We think the data in Fig.2a were recorded from a single neuron. We added the following sentence in the Result section (page 9).

Figure 2a shows a typical example of recordings from a single neuron.

The shape of action potentials was truncated because of strong depolarization. We added the following description in the Result section (page 10).

This phenomenon is interpreted that high-intensity oICMS induces large depolarization in cortical neurons, which lasts long after the cession of laser illumination (up to 10 ms), and truncates the shapes of action potentials because of depolarization block. These complex responses evoked by oICMS were also observed in eICMS (Supplementary Fig. 5a).

We did observe similar phenomena in eICMS (see the Supplementary Fig. 5a).

We also cited the reference 21: Herman et al. *Elife* **3**, e01481 (2014). They showed depolarization block by optogenetic stimulation.

8. Please provide details regarding how many neurons were recorded during optogenetic stimulation (and how many neurons were obtained from each monkey), and the proportion of neurons that were modulated by the stimulation. It would be useful to compare these proportions to those reported in other studies doing optogenetic stimulation of M1 in monkeys.

We showed this information in Supplementary Table 2.

9. Why use such very high frequencies (100 to 333 Hz) to drive ChR2? It is known that this opsin cannot follow such high frequencies (see Mattis et al 2012, Gunaydin et al. 2010). Were the neurons in cortex able to follow these frequencies? Were lower frequencies tested to see if those also induce movements?

The stimulus frequency of repetitive oICMS followed that was commonly used in repetitive eICMS (page 26).

oICMS at 333Hz also induced strong excitation with depolarization block (Fig. 3b).

We investigated the effect of stimulus frequency by observing evoked EMG and described as below (page 14-15). See also Fig. 4.

Concerning the frequency of repetitive stimulation, 333 Hz-stimulation was less effective in oICMS: The area of EMG by 333 Hz-oICMS was significantly smaller than those by 100 and 200 Hz-oICMS (*p<*0.0001 for 100 Hz and 200 Hz) (Fig. 4b, *left*), and also the peak amplitude by 333 Hz-oICMS was significantly smaller than that by 200 Hz-oICMS (*p<*0.0001) (Fig. 4b, *right*), suggesting that the effects of oICMS were saturated around 300 Hz.

10. Light intensities used to evoke movements cover a wide range (0.4 to 15 mW). Were all these equally effective, or was there an optimal intensity?

We added the following sentence (page 12).

Higher intensity of oICMS was more effective than lower intensity as in the case of eICMS.

11. The side by side comparison of electrical and optogenetic stimulation is one of the major strengths of this manuscript. It would be helpful to interpret the results to include some estimates of the extent of the area stimulated by one or the other method.

We compared the EMG evoked by oICMS and eICMS and added the following description (page 19).

Five-pulses oICMS induced similar amplitude of EMG as five-pulses eICMS did (Fig. 4), suggesting that both oICMS and eICMS activated the M1 to a similar extent.

We also recorded neuronal activity by oICMS and eICMS in Supplementary Fig. 5 and added the following sentence (page 10).

Both oICMS and eICMS induced excitation at a distance of 2.2 mm from the stimulation site (Supplementary Fig. 5b), suggesting that they both transsynaptically activated neurons in certain areas.

12. The electrical stimulation and optogenetic stimulation were delivered in the same location. Is it possible that the electrical stimulation ‘primed’ or ‘kindled’ the optogenetic stimulation? Were there controls in which only one the optogenetic stimulation was conducted?

This is not probable. Sometimes, we used only oICMS without eICMS, and observed evoked movements by oICMS. We did not see any threshold change of oICMS after eICMS.

13. The simultaneous recordings in M1 with EMG recordings are a great set of experiments. They rise a lot of important questions. It seems that the neuronal activity in M1 shown in figure 3a and 3b is, in fact, reduced during optogenetic stimulation (at least for the large unit). Is this so? Can a quantification of neuronal activity be provided to help assess this issue?

We added the following explanation (page 10).

This phenomenon is interpreted that high-intensity oICMS induces large depolarization in cortical neurons, which lasts long after the cession of laser illumination (up to 10 ms), and truncates the shapes of action potentials because of depolarization block. These complex responses evoked by oICMS were also observed in eICMS (Supplementary Fig. 5a).

We also cited the reference 21: Herman et al. *Elife* **3**, e01481 (2014). They showed depolarization block by optogenetic stimulation.

14. Along the same line, the results shown that the changes in the EMG traces evoked by 333 Hz is smaller than changes evoked by 100 and 200 Hz during optogenetic stimulation. This could be due to the inability of Chr2 to follow high frequencies.

We agree with the reviewer’s comment. We added the following sentence (page 15).

The effects of oICMS were saturated around 300 Hz.

15. Quantification of EMG responses should be better explained. For example in page 11, line 156, what does n=46 refers to? Same for line 164.

These numbers are the cortical sites where oICMS and eICMS were performed.

We added “sites” (page 13).

16. I recommend to plot the data in figure 4c and 4d as a bar graph instead of line-symbol graph. Also, I suggest to use SD instead of SEs in this graphs.

We changed Fig. 4 as suggested. See new Fig. 4.

17. In Discussion, it is stated that optogenetic responses were observed in layer 5. But, since the expression of the opsins seems to expand many layers, it is possible that the movements evoked by optogenetic stimulation were result of activation of neurons across cortical layers.

Thank you for your suggestion. It is probable. We added the following (page 16-17).

Our ingenious optrode could identify layer 5 cortical neurons expressing ChR2 and illuminate both superficial and deep cortical layers expressing ChR2 by the optical fiber.

18. Please include a table, or a description, of which AAV serotypes were injected in which monkeys, and which experiments were done in each (how many neurons obtained, how many EMG recordings made…).

We added Supplementary Tables 1 and 2.

19. In Methods, there is no description of the statistical analysis used.

We added the description in the Method section (page 29).

The area and peak amplitude of EMG were compared between different stimulus pulse numbers and between different stimulus frequencies by Friedman test with Dunn’s post hoc test. Significant level was set to *p*<0.05. Statistical analyses were performed using Prism software (GraphPad).

**Minor comments:**

• Page 9, line 128: ‘53/69 recording sites’. This data should be expressed as number of neurons recorded (multiple unit recordings could be spike sorted to obtain number of neurons)

We added “198/359 recording neurons, 55%” in page 10.

• Not very clear (at least to me) what is meant by ‘monotonously’ in page 12 line 186. Can other term be used to describe this response?

We deleted this sentence to avoid confusion.

• Are examples in figure 2 from the same neuron?

Figure 2a was recorded from the same neuron (Figure legend of Fig 2 in page 38).

The neuronal activity from one and the same neuron probably located in layer 5 was recorded, and thirty traces are overlaid.

Figure 2b was another neuron (Figure legend of Fig 2 in page 38)

Another example of M1 neuronal activity induced by single-pulse.

• In figures 2 and 3: In which layer of cortex were the neurons recorded?

Probably from neurons in layer 5 (as described in the Figure legends of Fig. 2 and 3).

• Discussion: The first paragraph is not really needed. It is just a recap of what optogenetic techniques are, and there are plenty of reviews that provide this information. I suggest to remove.

We removed most of the first paragraph of the Discussion section.

• Page 14, lines 219: Mention of the use of NpHR to modulate neurons in M1 of monkeys. A reference by Kinoshita et al 2012 is cited. The work by Diester et al 2011 should also be cited here.

We cited both Kinoshita et al 2012 and Diester et al 2011 (page 16).

• Page 16, line 245 “….AAV-DJ showed the most potent gene transfer”. As mentioned above, this very strong statement should be backed up by appropriate quantification.

We also analyzed quantitatively. Please see Supplementary Figs. 1c, 2ab, and 3

• Looking at figure 1, it seems that the tdTomato-containing vector was ‘less effective’ than the others. Again, a quantification could help assess if this is the case.

We agree with the reviewer’s comment and added the following sentence (page 7-8).

The area covered by hChR2(H134R)/EYFP signals (72.0 mm^2^, Monkey NR, Left M1) was larger than that by the tdTomato-containing vector (16.7 mm^2^, Monkey NR, Right M1).

• In Methods, it is stated that there was a 3 week waiting period between injecitons and the start of the optogenetic experiments. Perhaps I missed it, but I did not see the waiting period for the histology experiments to compare the various AAV serotypes (shown in fig 1)

We added the following (page 27-28).

After collection of enough data (in the case of electrophysiological experiments) or 2-4 weeks after AAV injections (for evaluation of AAV vectors), monkeys were deeply anesthetized with sodium pentobarbital.

• Stat symbols in figure 4c are unclear. The symbols do not seem to correspond with the legend. Please check.

We have corrected.

• If needed, were corrections for multiple comparisons done for the stats done in figure 4c?

We did multiple comparison test and described as follows (page 29).

The area and peak amplitude of EMG were compared between different stimulus pulse numbers and between different stimulus frequencies by Friedman test with Dunn’s post hoc test. Significant level was set to *p*<0.05. Statistical analyses were performed using Prism software (GraphPad).

• Was the histological examination conduced in all monkeys? Please clarify

Most of all. Please look at Supplementary Table 1.

**Reviewer #4 (Remarks to the Author):**

Watanabe et al. show that forelimb movements in monkeys can be evoked by optogenetic stimulation of motor cortex. Movements were similar to conventional electrical microstimualtion, except that there was slightly less non-linear summation. On one hand, these results are to be expected – optogenetics is widely used to activate neural circuits in rodents so it should not come as a surprise that activation of motor cortex, which projects directly to the spinal cord, should elicit movement. On the other hand, demonstrations of optogenetics in non-human primates are still relatively rare, with very few reports of behavioural effects, likely due to the larger brain size and the need to optimise viral delivery methods. Indeed, the difficulties of evoking motor potentials through optogenetics has been something of a barrier to adoption of optogenetics by the NHP neuroscience community, so it is good to see this problem resolved. Therefore demonstrating successful application of the
technique is an important contribution. However, it is a shame that there is little by way of new scientific insight contained within this paper, for example using the cell-specificity enabled by optogenetics to examine more about how motor cortical circuitry is engaged by oICMS and eICMS. In addition, there is little attempt to characterize stimulation effects, e.g. what area of cortex is activated, how many cells (as was undertaken for eICMS by studies such as Stoney et al (1968)).
Therefore, in my view the manuscript should be judged as a methods paper, and in this regard there are some important areas in which it falls short of the level of detail which would aid adoption by the community.

**Major comments:**
Choice of virus: An important part of getting optogenetics to work in NHPs is the choice of viral vector. Here the authors compare three AAV serotypes, in the end suggesting that AAV-DJ is best. This is extremely valuable data but no quantification is provided, only some example images. Quantification of the area of infected cortex, proportion of cells infected and what type of cells should be included. Although using a ubiquitous promotor, there may be differences in infection of different cell types - this can be looked at in histology data. In addition, it would be useful to know if corticospinal tract and corticomotoneuronal cells are infected - this could be observed in spinal cord histology.

We added Supplementary Figs. 1, 2, and 3 and the following description (page 6-7).

The fluorescence signals were most conspicuous around the injection sites of the AAV-DJ vector (Supplementary Fig. 1b). The areas and number of cells transduced by the AAV-DJ vector were larger than those transduced by AAV2 and AAV5 (Supplementary Fig. 1c). The intensity of fluorescence signals at the single-neuron level by the AAV-DJ vector was higher than that by AAV2 and AAV5 (Supplementary Fig. 2a). Observing expression of NeuN, parvalbumin (PV), and glial fibrillary acidic protein (GFAP) among EGFP positive cells (Supplementary Fig. 2b, c) showed that the major cell type expressing EGFP was NeuN-positive neurons, and that glial cells expressing EGFP were a small population (Supplementary Fig. 2b). The AAV-DJ vector induced EGFP expression at terminals in the putamen and at axons presumably in the lateral corticospinal tract of the spinal cord, but no retrogradely labeled cells were found in the thalamus (Supplementary Fig. 2d). In addition, the EGFP protein level induced by the AAV-DJ vector in the mouse brain was higher than that induced by AAV2 and AAV5 (Supplementary Fig. 3).

Optrode: The authors describe a new optrode for delivering light to the tissue, but there is no attempt to compare with existing commercial designs, or provide motivation for the particular design choices made for this study. In addition, optical power is given in mW, measured 5mm from the optrode tip. However, the separation between optrode tip and recording site is about 1mm. The authors should provide details on light intensity measured as power per unit area at the electrode tip - this could then be related to thresholds for opsin activation. This would require some consideration of the light spread from the fibre, but would allow easier comparison with other papers in the literature.

We can electrically stimulate using this optrode, and such an optrode is not commercially available (page 8).

We made an optrode by bundling 1) a glass coated tungsten electrode for recording, 2) an optical fiber for optical stimulation, and 3) a pair of tungsten wires for bipolar electrical stimulation (Fig.1d, see Methods for details). This optrode enabled us to record neuronal activity induced by optical and electrical stimulation.

We measured at of distance 1 mm and expressed as mW/mm^2^ as suggested (page 25).

Light power was measured in mW using a power and energy meter (Ophir Optronics) at a distance of 1 mm from the light-emitting tips and calculated as power per unit area (mW/mm^2^).

The truncated spikes occurring after stimulation should be better documented. These bursts appear unusual (e.g. compare with Diester et al. Nat Neurosci 2011 where spikes are elicited only during stimulation) and look a bit like injury potentials – is there a chance that the neuron could be unhealthy and/or damaged by the optrode? A figure with overlaid spikes, to convince readers that they are actually from the same neuron would help. For how long are they truncated? Is the firing pattern of these neurons normal when not stimulating, or do these cells generally fire in a burst manner (which could be an indication of injury).

We added the following explanation (page 10).

This phenomenon is interpreted that high-intensity oICMS induces large depolarization in cortical neurons, which lasts long after the cession of laser illumination (up to 10 ms), and truncates the shapes of action potentials because of depolarization block. These complex responses evoked by oICMS were also observed in eICMS (Supplementary Fig. 5a).

We also cited the reference 21: Herman et al. *Elife* **3**, e01481 (2014). They showed depolarization block by optogenetic stimulation.

We added the following in the legend of Fig 2 (page 38).

The neuronal activity from one and the same neuron probably located in layer 5 was recorded, and thirty traces are overlaid.

We do not think they are injury potentials because of the following as described in the legend of Fig. 2 (Page 38).

Note that no spikes were observed before stimulation, suggesting that the recording neuron was not injured by the optrode.

**Minor comments:**
Line 117: Could the deflection observed when an optogenetic stimulation is delivered be a photoelectric effect? A common control is to use a light with a different wavelength. Instead it is stated that the deflection was seen “observed only at the areas where ChR2 was expressed” but this important point is not shown or quantified in the paper.

We added the following description (page 9-10). See also Supplementary Fig. 4

The deflection is unlikely an artifact, because (1) it was not induced by a 589 nm yellow laser light, and (2) it was not observed at non-injection sites (Supplementary Fig. 4).

Line 153: the authors should specify if the EMG responses were recorded whilst the primate was performing any specific behaviour.

We added the following sentence (page 27).

Body movements and electromyogram (EMG) were observed when monkeys were at rest without any movements

Line 185: oICMS shows lower temporal summation than eICMS. This refers to figure 4. There may be another explanation in that single pulse oICMS is produces a response that is three time higher than single pulse eICMS. Therefore it is possible that the response to extra stimulation pulses is saturated.

We deleted the description on temporal summation and described as (page 14):

These results suggest that oICMS induces muscle activity more effectively than eICMS at fewer pulses.

Methods: Please state which muscles were recorded from.

We added the following sentence (page 27).

Although body parts in which movements and/or EMG were evoked depended on the cortical site of oICMS/eICMS, EMG of the extensor carpi radialis longus and flexor carpi radialis muscles was mainly recorded and used for further analyses.

In Fig. 3, EMG was recorded from the extensor carpi radialis longus (Extensor) and flexor carpi radialis (Flexor) muscles (Figure legend of Fig. 3, page 39).

In Fig.4, Raw EMG recorded from the flexor carpi radialis muscle (Figure legend of Fig. 4, page 39).

**Reviewer comments, second version:**

Reviewers' comments:

Reviewer #3 (Remarks to the Author):

In this revised version of the manuscript, Watanabe et al. addressed many of the concerns and comments raised in the first submission. The revised version strengthened my view that this will be an important paper to support further use of optogenetics in non-human primates. The new supplementary material and tables are very useful. In my view, however, there remain some points that require additional clarification, listed as follows.
• The side by side comparison of the different serotypes will provide relevant information for future studies, but some additional details and clarifications are needed. Supp. Fig. 1, panel c: The quantification of the area transduced by the viral vectors is, likely, confounded by the amount of virus injected. In other words, it is not surprising that AAV-DJ covers a larger area than the other two serotypes, because in most cases, a larger amount of virus was injected with this serotype (in average, the amount of virus injected was 3, 4.5 and 7.25 ul for AAV2, 5 and DJ respectively). Also, there are many more cases of DJ injections than AAV2 and AAV5. Based on this, the results should be normalized (for example, per microl injected).
• Other comments about the histologic analysis:
o How many cells (total per animal) were used for the quantification of the fluorescence intensity per cell?
o Supplemental fig. 2b: The legend states “Percentages of NeuN (marker for neuron), palvalbumine (PV, marker for PV containing interneurons), and GFAP (markers for glial cells) positive cells among EGFP positive cells” It is unclear if the percentages indicated are for all NeuN-positive cells that express GFP, or for all GFP cells that express NeuN. If the latter, the added percentages of EGFP-positive cells transduced with AAV5 and AAVDJ add to more than 100%. Please clarify.
o In supplemental material, lines 31-32: “For measuring the extent of the areas covered by EGFP positive cells, hChR2(H134R)/EYFP or hChR2(H134R)/tdTomato signals were manually surrounded and measured using ImageJ software..”. Please indicate how many sections were used to measure this area, of if the area was defined based on the section with the largest extent of transduction
o In the rebuttal letter, the authors state that there was no overlap of GFP expression in the brain tissue of monkeys that received more than one type of viral vector. This statement should be included in the manuscript.
o Please indicate which antero-posterior levels are shown in figure 2d images, and specify which segment of VL is shown (VLa or VLp)
• In my opinion, the graphs in fig. 4 remain challenging to understand. The main point (to show that oICMS is generally more effective than eiCMS to induce EMG responses) gets lost among all the comparisons. I wonder if one of the two measurements (Area of EMG or Peak amplitude of EMG) could be shown as a supplementary figure, so that the main figure could be expanded or presented differently. For example, one panel could show the comparisons among oICMS conditions, another panel display comparisons among eICMS, and a third panel would show comparisons among oICMS and eICMS (perhaps consolidating/averaging results obtained per frequency)
• The control experiments shown in Supp. Fig 4b greatly strengthen the results in this paper. Please describe in how many sites (and how many monkeys) were these control experiments conducted.
• Please include in Supp Table 1 the number of injection tracks done in each animal.
• In most studies, the titer of viral vectors is expressed in vg/ml (not vg or particles/microl, see, for example, Gray et al, 2011, Curr. Protoc. Neurosci). I strongly suggest to express the titer using the ml convention. This will make it easier for the reader to compare this study to previous published papers. In most NHP optogenetic studies that have used AAVs, the titers ranged from 10e12 to 10e13 particles/ml.
• Was the titer of virus solution the same (1.5x10e9) for AAV2-CAG-EGFP, AAV5-CAG-EGFp and AAVDJ-CAG-EGFP (Methods, page 23)? Please clarify in the text.
• Regarding the intensities of light used in this study, the authors state that: “Higher intensity of laser (51-1910 mW/mm2) was used for illumination than in previous studies (3-1600 mW/mm2) “ (page 16). This statement, per se, does not seem to support that the light intensities used in this study were significantly higher than in previous studies, because these ranges of light (3-1600 and 51-1910) largely overlap. However, later in the manuscript (page 18) it is stated: “Previous studies targeting M1 cortical neurons expressing ChR2 used weaker laser (3-255 mW/mm2) “. I suggest to keep the latter statement (in page 18) and discard the first (page 16); since the statement in page 18 is directly relevant to this study (comparison of the current experiment to others in which optogenetic stimulation was done in M1 in monkeys). The statement in page 16, instead, compares this study to the study by Cavanaugh et al, in which the optogenetic manipulation was done in V1.
• In page 18 “…expressing ChR2 used weaker laser” This should be rephrased. Previous studies did not use weaker lasers, but lower light intensities. Along the same line, I suggest to replace “intensity of laser” for “intensity of light” or “intensity of illumination” throughout the manuscript
• In response to my previous question if the optical stimulation could somehow ‘prime’ for optogenetic stimulation, the authors replied (in rebuttal letter): “This is not probable. Sometimes, we used only oICMS without eICMS, and observed evoked movements by oICMS. We did not see any threshold change of oICMS after eICMS.” This statement should be included in the manuscript.
• Line 131 “Stronger oICMS…” should be replaced by ‘ longer and repeated pulses of oICMS”, since bottom trace in figure 2a is the same intensity as the second trace, but twice as long and given as a train of 100 Hz
• How were the multiple recording experiments (fig 5b) conducted? Using a second microdrive? Please describe
• Lines 154-155: “The inhibition may be caused by inhibitory cortical interneurons expressing ChR2 under the control of a ubiquitous CAG promoter. “ This is not likely, based on the results of the histology (Supp. Fig 2b) Please comment on this.
• Lines 268-270 should be rephrased or removed. It is not clear what is the point of discussing the efficacy of AAV2, particularly if this statement is preceded by another, at the beginning of the paragraph, indicating that optogenetics have been successfully deliver in NHP brains.
• The description of results in figure 2 remains incomplete and anecdotal. How many neurons presented a similar response to the one shown in figure 2? The question if the spikes correspond to more than one unit remains unanswered, and the authors do not comment if the multi-unit excitation and complex responses are related to the evoked movement, or they are just an epiphenomenon


Reviewer #5 (Remarks to the Author):

In my view this is a method paper as no new scientific insight on how the motor cortex is engaged by oICMS has been demonstrated. These insights could have been gained by taking advantage of the optogenetic power to target specific groups of cells. This has been prevented by the use of a ubiquitous promotor.

In the supplementary figure 1 the authors have done a quantification of the cells in M1 that have been transfected and also compared the area and the intensity of the fluorescence in M1. However I am concerned by the proximity of the injections sites from the three viruses. There will be diffusion of the virus, so how certain are the authors that the cells at each site were only transfected by the injected virus. Could some cells have been transfected by a combination of viruses? Again the use of the same reporter (EGFP) makes it hard to discriminate between the contributions of the different viruses.

The paper is the first to achieve limb movements with oICMS, therefore a more careful characterisation of the spread of the stimulation was expected. The authors provide a light power per unit area, however this is not sufficient as it doesn’t take into account the light scattering in the brain tissue. The oICMS is a relatively new method so it needs a characterisation that could have been achieved by taking advantage of the existence of corticospinal neurons in M1 that can be activated and identified by stimulation of the pyramidal tract. A proper diagram showing the intensity of the stimulation and the area covered in the brain tissue would have provided a better characterisation of this virus and of the oICMS in the motor cortex.

The projections of the transfected neurons to the spinal cord are poorly estimated. The main point of this paper is the ability to induce movements by using optogenetics and this certainly has to go through the corticospinal tract. Is the AAV-DJ more potent in transfecting corticospinal neurons or corticomotoneuronal cells? This could have been achieved by comparing the projections in the spinal cord terminals of the transfected CST neurons. A proper histology of these projections in the ventral horns could have given proper insights on how this particular virus was efficient in evoking twitches. The use of the same reporter and simultaneous injections of AAV2, AAV5 and AAV-DJ in the same monkey make this comparisons between the three viruses at the level of the spinal cord very difficult. Therefore the figure 2 in the supplementary is not convincing. The authors have also looked at the projection to or from the Putamen and the Thalamus, however there is no proper explanation on why these structures have been particularly looked at.

The sentence stipulating that oICMS is more efficient than eICMS is not convincing as this solely relies on the comparisons of the single shocks. But the intensity of the two stimulations should have been matched to get the same EMG response with single shocks. Then the effects of temporal summation evoked by the two types of stimulation when delivering two or three shocks could have been compared.

The probe in itself is not an innovation. The authors insist that this material is not commercially available. However Neuronexus, Plexon and Microprobe all deliver probes and arrays containing fiber optics, multiples contacts – some can be used for stimulations others for recording. With the lengths and distances between the contacts that can be adjusted and smaller to the probe the authors have designed. Therefore the probe is not a new method.

Minor comments:
The authors statistical claim is not correct
"220 The area and peak amplitude by one and two pulses of eICMS
221 were similar to each other (p>0.05), and those by two and three pulses of eICMS were
222 also similar to each other (p>0.05). "

The fact that P>0.05 doesn’t prove that the effect of 2 or 3 shocks stimulations were similar to each other. The authors can not use this test to conclude that the two compared types of stimulation had similar effects. The only conclusion they can gather from P>0.05 is that they didn’t manage to reject the hypothesis stipulating that the two stimulation had similar effects.

"228 effects of oICMS were saturated around 300 Hz."
Why does 333HZ induces smaller effects than 100 and 200 Hz? If oICMS was saturating at 333Hz why the effect was not just as big as with the smaller frequencies. At the same time higher frequency seems to induce a higher effect on movements. What could be the mechanisms that could explain a smaller effects on EMG when applying a higher frequency – especially that these effect don’t seem to happen with eICMS (3 shocks at 333 gave bigger effects than at 100).

**Author rebuttal, second version:**

Reviewer #3 (Remarks to the Author):

In this revised version of the manuscript, Watanabe et al. addressed many of the concerns and comments raised in the first submission. The revised version strengthened my view that this will be an important paper to support further use of optogenetics in non-human primates. The new supplementary material and tables are very useful. In my view, however, there remain some points that require additional clarification, listed as follows.

• The side by side comparison of the different serotypes will provide relevant information for future studies, but some additional details and clarifications are needed. Supp. Fig. 1, panel c: The quantification of the area transduced by the viral vectors is, likely, confounded by the amount of virus injected. In other words, it is not surprising that AAV-DJ covers a larger area than the other two serotypes, because in most cases, a larger amount of virus was injected with this serotype (in average, the amount of virus injected was 3, 4.5 and 7.25 ul for AAV2, 5 and DJ respectively). Also, there are many more cases of DJ injections than AAV2 and AAV5. Based on this, the results should be normalized (for example, per microl injected).

Thank you for your helpful suggestion. The area transduced by the viral vectors was normalized to the amount of viral vector injected in **Supplementary Fig. 1c** and in the manuscript.

(**main text, page 8**)

The area covered by hChR2(H134R)/EYFP signals (18.0 mm^2^/µl injected, Monkey NR, Left M1) was larger than that by the tdTomato-containing vector (4.2 mm^2^/µl injected, Monkey NR, Right M1).

• Other comments about the histologic analysis:
• How many cells (total per animal) were used for the quantification of the fluorescence intensity per cell?

We added the number of cells used in **Supplementary Fig. 2a**.

• Supplemental fig. 2b: The legend states “Percentages of NeuN (marker for neuron), parvalbumin (PV, marker for PV containing interneurons), and GFAP (markers for glial cells) positive cells among EGFP positive cells” It is unclear if the percentages indicated are for all NeuN-positive cells that express GFP, or for all GFP cells that express NeuN. If the latter, the added percentages of EGFP-positive cells transduced with AAV5 and AAVDJ add to more than 100%. Please clarify.

Sorry for the inconvenience. The percentages indicated are for all EGFP-positive cells that express NeuN, PV or GFAP. PV-positive cells are neurons, therefore NeuN-positive cells also include PV-positive cells. This is the reason why the added percentages of EGFP-positive cells are more than 100%.

• In supplemental material, lines 31-32: “For measuring the extent of the areas covered by EGFP positive cells, hChR2(H134R)/EYFP or hChR2(H134R)/tdTomato signals were manually surrounded and measured using ImageJ software..”. Please indicate how many sections were used to measure this area, of if the area was defined based on the section with the largest extent of transduction.

We added the number of slices used in Supplementary Information.

(**Supplementary Information, page 2**).

For measuring the extent of the areas covered by EGFP-positive cells transduced with AAV2 (examined in 8 sections), AAV5 (10 sections), or AAV-DJ (12 sections), and hChR2(H134R)/EYFP (13 sections) or hChR2(H134R)/tdTomato (6 sections) signals were manually surrounded and measured using ImageJ software (National Institutes of Health).

• In the rebuttal letter, the authors state that there was no overlap of GFP expression in the brain tissue of monkeys that received more than one type of viral vector. This statement should be included in the manuscript.

Thank you for your good suggestion. We added the following statement in the manuscript. (**main text, page 6**).

Each injection site was separated at least 2 mm (Supplementary Fig. 1a), and there was no overlap of EGFP expression transduced by each serotype of viral vector in the brain sections.

• Please indicate which antero-posterior levels are shown in figure 2d images, and specify which segment of VL is shown (VLa or VLp)

Thank you for your helpful suggestion. VL in Supplementary Fig. 2d corresponds to VLp, therefore we changed VL to VLp in Fig. 2d and figure legend.

• In my opinion, the graphs in fig. 4 remain challenging to understand. The main point (to show that oICMS is generally more effective than eiCMS to induce EMG responses) gets lost among all the comparisons. I wonder if one of the two measurements (Area of EMG or Peak amplitude of EMG) could be shown as a supplementary figure, so that the main figure could be expanded or presented differently. For example, one panel could show the comparisons among oICMS conditions, another panel display comparisons among eICMS, and a third panel would show comparisons among oICMS and eICMS (perhaps consolidating/averaging results obtained per frequency)

Thank you for your helpful suggestion. We have changed **Fig. 4** and made new **Supplementary Fig. 6**. In **Fig 4b**, the area of EMG evoked by oICMS and eICMS is compared. The relative area of EMG is also shown in **Fig. 4c**. In **Supplementary Fig. 6**, the peak amplitude of EMG evoked by oICMS and eICMS is compared.

• The control experiments shown in Supp. Fig 4b greatly strengthen the results in this paper. Please describe in how many sites (and how many monkeys) were these control experiments conducted.

We have added the numbers of sites and monkeys used for the control experiments.

**(**figure legend of **Supplementary Fig. 4**)

Similar recordings were obtained in 13 sites in Monkey PT and 15 sites in Monkey MG.

• Please include in Supp Table 1 the number of injection tracks done in each animal.

We have added the numbers of injection tracks and sites in **Supplementary Table 1**.

• In most studies, the titer of viral vectors is expressed in vg/ml (not vg or particles/microl, see, for example, Gray et al, 2011, Curr. Protoc. Neurosci). I strongly suggest to express the titer using the ml convention. This will make it easier for the reader to compare this study to previous published papers. In most NHP optogenetic studies that have used AAVs, the titers ranged from 10e12 to 10e13 particles/ml.

Thank you for your practical suggestion. We changed the titer using the ml convention in the manuscript (**main text, page 6 and 24**; **Supplementary Information, page 2**).

• Was the titer of virus solution the same (1.5x10e9) for AAV2-CAG-EGFP, AAV5-CAG-EGFP and AAVDJ-CAG-EGFP (Methods, page 23)? Please clarify in the text.

Sorry for the inconvenience. The titers of AAV2-CAG-EGFP, AAV5-CAG-EGFP, and AAV-DJ-CAG-EGFP were the same (1.5 x 10e12 vg/ml). We clarified this in the manuscript (**main text, page 6 and 24**).

• Regarding the intensities of light used in this study, the authors state that: “Higher intensity of laser (51-1910 mW/mm2) was used for illumination than in previous studies (3-1600 mW/mm2) “ (page 16). This statement, per se, does not seem to support that the light intensities used in this study were significantly higher than in previous studies, because these ranges of light (3-1600 and 51-1910) largely overlap. However, later in the manuscript (page 18) it is stated: “Previous studies targeting M1 cortical neurons expressing ChR2 used weaker laser (3-255 mW/mm2) “. I suggest to keep the latter statement (in page 18) and discard the first (page 16); since the statement in page 18 is directly relevant to this study (comparison of the current experiment to others in which optogenetic stimulation was done in M1 in monkeys). The statement in page 16, instead, compares this study to the study by Cavanaugh et al, in which the optogenetic manipulation was done in V1.

Thank you for your helpful suggestions. According to the suggestions, we changed the manuscript as follows.

(**main text, page 17**)

Intensity of laser light (51-1910 mW/mm^2^) used for illumination was stronger than that in previous studies targeting the M1 (3-255 mW/mm^2^)^12, 13, 15^.

(**main text, page 18-19**)

In the present study, we used high intensity of laser light (51-1910 mW/mm^2^) compared to previous studies. Previous studies targeting M1 cortical neurons expressing ChR2 used weaker laser light (3-255 mW/mm^2^)^13, 14^ and could not affect behaviors. On the other hand, eye movements were successfully affected by higher-intensity laser light. Optogenetic inactivation of superior collicular (SC) neurons expressing Archaerhodopsin-T using green laser light (650-1600 mW/mm^2^) shifted saccadic end point, reduced peak velocity, and increased latency^12^. Optogenetic activation of ChR2-expressing terminals in the SC using blue laser light (< 1100 mW/mm^2^)^10^ , excited SC neuron, and evoked saccadic eye movements. Thus, we decided to use higher-intensity laser light in this study.

• In page 18 “…expressing ChR2 used weaker laser” This should be rephrased. Previous studies did not use weaker lasers, but lower light intensities. Along the same line, I suggest to replace “intensity of laser” for “intensity of light” or “intensity of illumination” throughout the manuscript

We have changed “laser” to “laser light” throughout the manuscript.

• In response to my previous question if the optical stimulation could somehow ‘prime’ for optogenetic stimulation, the authors replied (in rebuttal letter): “This is not probable. Sometimes, we used only oICMS without eICMS, and observed evoked movements by oICMS. We did not see any threshold change of oICMS after eICMS.” This statement should be included in the manuscript.

Thank you for your good suggestion. We have added the statement in the manuscript.

(**main text, page 19**)

Sometimes, we used only oICMS without eICMS, and observed evoked movements by oICMS. We also examined motor threshold of oICMS before and after eICMS, and did not see any threshold changes, suggesting no priming effects of eICMS on oICMS.

• Line 131 “Stronger oICMS…” should be replaced by ‘ longer and repeated pulses of oICMS”, since bottom trace in figure 2a is the same intensity as the second trace, but twice as long and given as a train of 100 Hz

We have changes “stronger” to “Longer and repetitive” according to the suggestion.

(**main text, page 9-10**)

Longer and repetitive oICMS induced repetitive directly evoked spikes (* in the bottom trace of Fig. 2a, 0.9 mW, 2 ms).

• How were the multiple recording experiments (fig 5b) conducted? Using a second microdrive? Please describe

We have added the following description.

(figure legend of **Supplementary Fig. 5**)

Neuronal activity recorded at a horizontal distance of 2.2 mm from the oICMS/eICMS site using another recording electrode, which was inserted into the M1 using the second micromanipulator.

• Lines 154-155: “The inhibition may be caused by inhibitory cortical interneurons expressing ChR2 under the control of a ubiquitous CAG promoter. “ This is not likely, based on the results of the histology (Supp. Fig 2b) Please comment on this.

Thank you for your good suggestion. We have changed the description as follows.

(**main text, page 11**)

One possible origin of the inhibition is inhibitory cortical interneurons expressing hChR2; however, this is unlikely, because PV-positive inhibitory cortical interneurons were less labeled with EGFP (Supplementary Fig. 2b).

• Lines 268-270 should be rephrased or removed. It is not clear what is the point of discussing the efficacy of AAV2, particularly if this statement is preceded by another, at the beginning of the paragraph, indicating that optogenetics have been successfully deliver in NHP brains.

We have deleted this sentence related to AAV2.

(**main text, page 17**)

AAV vectors are the most widely applied tools in optogenetics and have been successfully used to deliver opsins into the mouse, rat, and non-human primate brains. Previous studies evaluated transduction efficiency of different serotypes of AAV vectors, i.e., 1, 2, 5, 8, and 9, in the cortex of macaque monkeys^30, 31^.

• The description of results in figure 2 remains incomplete and anecdotal. How many neurons presented a similar response to the one shown in figure 2?

We have added the number of recording sites where truncated action potentials, sign of strong excitation, were observed.

(**main text, page 10**).
Such truncated action potentials were observed at 55% (104/190 sites) of the sites where oICMS induced excitation.

The question if the spikes correspond to more than one unit remains unanswered, and the authors do not comment if the multi-unit excitation and complex responses are related to the evoked movement, or they are just an epiphenomenon

The multiunit activity as observed in Fig. 2 does not directly evoked movements. But, truncated action potential due to depolarization block and multiunit activity in Fig. 2 are the sign of strong activation of the cortex, which evokes movements. Thus, we added the following sentence.

(**main text, page 11**)

The data in Fig. 2a, b indicate that oICMS effectively activates M1 neurons.

Reviewer #5 (Remarks to the Author):

In my view this is a method paper as no new scientific insight on how the motor cortex is engaged by oICMS has been demonstrated. These insights could have been gained by taking advantage of the optogenetic power to target specific groups of cells. This has been prevented by the use of a ubiquitous promotor.

In the supplementary figure 1 the authors have done a quantification of the cells in M1 that have been transfected and also compared the area and the intensity of the fluorescence in M1. However I am concerned by the proximity of the injections sites from the three viruses. There will be diffusion of the virus, so how certain are the authors that the cells at each site were only transfected by the injected virus. Could some cells have been transfected by a combination of viruses? Again the use of the same reporter (EGFP) makes it hard to discriminate between the contributions of the different viruses.

Thank you for the suggestion. We added the following statement in the manuscript to avoid confusion.

(**main text, page 6**)

Each injection site was separated at least 2 mm (Supplementary Fig. 1a), and there was no overlap of EGFP expression transduced by each serotype of viral vector in the brain sections.

The paper is the first to achieve limb movements with oICMS, therefore a more careful characterisation of the spread of the stimulation was expected. The authors provide a light power per unit area, however this is not sufficient as it doesn’t take into account the light scattering in the brain tissue. The oICMS is a relatively new method so it needs a characterisation that could have been achieved by taking advantage of the existence of corticospinal neurons in M1 that can be activated and identified by stimulation of the pyramidal tract. A proper diagram showing the intensity of the stimulation and the area covered in the brain tissue would have provided a better characterisation of this virus and of the oICMS in the motor cortex.

There are many studies on the scattering of the laser light in the brain tissue. We cited Ozden et al. (2013) and discussed effective area of light stimulation based on their experimental and simulation data.

(**main text, page 20**)

One reason is that oICMS illuminates neural tissue along the long axis and effectively activates layer 5 pyramidal neurons. Based on the study by Ozden et al., 2013^40^, 1 mW of light through an 200-µm optical fiber sheds 1 mW/mm^2^ light inside a spheroidal area, whose size is 1 mm in the axial direction and 0.3 mm in the radial direction (for details, see Fig. 4a in Ozden et al., 2013^40^). Inside this area, neurons expressing ChR2 are expected to be activated. When the optrode is inserted into the anterior bank of the central sulcus, laser light sheds layer 5 from the side and can activate layer 5 pyramidal neurons over a wide area.

The projections of the transfected neurons to the spinal cord are poorly estimated. The main point of this paper is the ability to induce movements by using optogenetics and this certainly has to go through the corticospinal tract. Is the AAV-DJ more potent in transfecting corticospinal neurons or corticomotoneuronal cells? This could have been achieved by comparing the projections in the spinal cord terminals of the transfected CST neurons. A proper histology of these projections in the ventral horns could have given proper insights on how this particular virus was efficient in evoking twitches. The use of the same reporter and simultaneous injections of AAV2, AAV5 and AAV-DJ in the same monkey make this comparisons between the three viruses at the level of the spinal cord very difficult. Therefore the figure 2 in the supplementary is not convincing. The authors have also looked at the projection to or from the Putamen and the Thalamus, however there is no proper explanation on
why these structures have been particularly looked at.

We observed the spinal cord, putamen and thalamus based on the comments by previous reviewers: It would be useful to know if corticospinal tract and corticomotoneuronal cells are infected - this could be observed in spinal cord histology (Reviewer #4), The results should include a description of anterograde and retrograde transport of the opsins (Reviewer #3).

We added Supplementary Fig. 2d and the following description in the manuscript.

(**main text, page 7**)

The projection sites of the M1 were also examined. The AAV-DJ vector induced EGFP expression at terminals in the putamen and at axons presumably in the lateral corticospinal tract of the spinal cord, but no retrogradely labeled cells were found in the thalamus (Supplementary Fig. 2d).

The sentence stipulating that oICMS is more efficient than eICMS is not convincing as this solely relies on the comparisons of the single shocks. But the intensity of the two stimulations should have been matched to get the same EMG response with single shocks.

We added new Fig. 4c. This figure shows the relative area of EMG, which was normalized to that evoked by single-pulse oICMS/eICMS.

We also changed the description “oICMS is more efficient than eICMS” as following.

(**main text, page 14**)

These results suggest that repetitive oICMS induces muscle activity effectively as repetitive eICMS does.

Then the effects of temporal summation evoked by the two types of stimulation when delivering two or three shocks could have been compared.

We compared oICMS and eICMS in Fig. 4c showing normalized EMG and described as follows.

(**main text, page 15**)

Similar tendency was observed in the relative area of EMG evoked by repetitive oICMS/eICMS (Fig. 4c). The relative area of EMG evoked by oICMS increased as the numbers of stimulus pulses increased only at 100 Hz, whereas that by eICMS exponentially increased as the number of stimulus pulses increased at 100, 200, and 333 Hz, suggesting that oICMS shows less temporal summation than eICMS.

The probe in itself is not an innovation. The authors insist that this material is not commercially available. However Neuronexus, Plexon and Microprobe all deliver probes and arrays containing fiber optics, multiples contacts – some can be used for stimulations others for recording. With the lengths and distances between the contacts that can be adjusted and smaller to the probe the authors have designed. Therefore the probe is not a new method.
We agreed with the reviewer’s opinion. We simply describe as a homemade electrode.

Minor comments:
The authors statistical claim is not correct
"220 The area and peak amplitude by one and two pulses of eICMS
221 were similar to each other (p>0.05), and those by two and three pulses of eICMS were
222 also similar to each other (p>0.05). "
The fact that P>0.05 doesn’t prove that the effect of 2 or 3 shocks stimulations were similar to each other. The authors can not use this test to conclude that the two compared types of stimulation had similar effects. The only conclusion they can gather from P>0.05 is that they didn’t manage to reject the hypothesis stipulating that the two stimulation had similar effects.

Thank you for your comments. According to the suggestion, we have changed the description throughout the manuscript.

"228 effects of oICMS were saturated around 300 Hz."
Why does 333HZ induces smaller effects than 100 and 200 Hz? If oICMS was saturating at 333Hz why the effect was not just as big as with the smaller frequencies. At the same time higher frequency seems to induce a higher effect on movements. What could be the mechanisms that could explain a smaller effects on EMG when applying a higher frequency – especially that these effect don’t seem to happen with eICMS (3 shocks at 333 gave bigger effects than at 100).
We changed the description and added discussion as follows.

(**main text, page 15**)

the effects of oICMS were saturated and became smaller at 333 Hz.

(**main text, page 21**)

The effects of oICMS at 333 Hz were less effective than those at 100 and 200 Hz. This is probably due to photocycle kinetics of hChR2: fast channel opening, but relatively slow channel closing and slow recovering to the ground state^42, 43^. Actually, during high-frequency optical stimulation, stimulus pulses often fail in inducing action potentials^44, 45^

**Reviewer comments, third version:**

Reviewer #3:
Remarks to the Author:
The revised version of the manuscript has addressed many of the concerns I expressed to the second submission. However, there are still some points that need to be clarified.
• The authors claim there was no overlap of the EGFP expression among the injections performed. However, this is difficult to judge based on the example images provided. Supplementary figure 1b (third row) shows a potential overlap between the 2 tracks indicated. It would be helpful if the authors can add some information about how the extent of one injection was defined, and how they concluded there was no overlap.
• Please provide higher magnification of the areas with presumed GFP+ terminals (panel D in supplementary figure 2). In the text, it should be mention that the areas shown (putamen, thalamus and lateral corticospinal tract) show putative axon terminals (with the available data, it cannot be firmly concluded that these are axon terminals).
• It is suggested that the ‘truncated shapes’ of the action potentials are due to depolarization block. This remains speculative. To back up this statement an intracellular study is referenced (ref. 20, Herman et al 2014). It would be more relevant to the current study if the authors provide references of studies showing depolarization block during extracellular recordings.
• In the experiments looking at the distant effects of oICMS and eICMS the distance between the stimulation and recording sites is 2.2 mm (Supplementary fig. 5b). How was this distance chosen? Were other distances examined? It would be of great interest to know at what distance the effects disappear.
• Based on the histology observations, it is unlikely that PV-positive interneurons were responsible for the inhibitory responses (lines 160-161). This does not rule out, however, that PV-negative inhibitory neurons may have expressed the ChR2 and played a role in the inhibitions.
• Please rephrase “…were decreased more slowly…” (line 207). I believe it would be more appropriate to say that the area and peak amplitude obtained by oICMS did not show a corresponding decrease with the number of stimulus pulses.
• How was the “Relative area of EMG” obtained? This should be explained in methods.
• Please clarify how layer 5 neurons were identified in electrophysiological recordings (lines 265, 742 and 748). If the neurons were putatively identified as layer 5 neurons (as suggested in lines 742 and 748) this should be made clear throughout the manuscript.
• I suggest to use only ‘light intensity’ instead of ‘laser light intensity’ throughout the manuscript. ‘Laser light’ seems redundant.
• In Discussion, lines 285 through 286 (list of other excitatory opsins) seems irrelevant. I suggest removing this part.
• The wording “laser light sheds layer 5 from the side and can activate layer 5 pyramidal neurons over a wide area” (lines 326-237) is awkward. Can this be reworded?
• In Discussion the authors state that “during high-frequency optical stimulation, stimulus pulses often fail in inducing action potentials: (lines 335 and 336). This statement contradicts the results, “Figure 2b also shows that action potentials followed each stimulus pulse at 200 Hz (Fig. 2b, 15 mW corresponding to 1910 mW/mm2, 2 ms, 200 Hz, 10 pulses” (lines 163-164). Please clarify.
• Line 60 “stronger light”. Please clarify this means stronger light intensity than in previous studies.
• In supplementary figure 1b, please indicate from which area of the low magnification image was the high magnification image obtained.


Reviewer #5:
Remarks to the Author:
The authors have changed their manuscript and figures and have addressed many of the concerns. This has made their article stronger. I still think this is a method paper that demonstrates for the first time, that optogenetic stimulation of motor cortex in non-human primate can be achieved with the use of a specific virus. This can be of a great interest to the scientific community. The spiking activity in fig 2 is worth documenting by itself as this kind of responses are not visible with eICMS because of the large artefact that electrical stimulation will induce. This is one advantage of the oICMS that is worth discussing. However the characterisation of the cells recruited by the oICMS to achieve this motor responses has not been fully considered specially regarding the corticospinal tract neurons. Also I have concerns regarding the interpretation of the results shown in figure 4.


Can the image on the figure2d in the supplementary data for the corticospinal tract be zoomed out to see that it is the lateral corticospinal tract in the spinal cord and zoomed in so we can see clearly the expression of the EGFP?
The expression of the virus in the tract will demonstrate that the motor effect induced by oICMS in M1 is due partly to direct stimulation of the corticospinal tract, even though this link is hard to establish because three different viruses were injected in the same animal which makes it difficult to distinguish which virus has transfected the corticospinal neurons.


The statement that "repetitive oICMS induces muscle activity effectively as repetitive eICMS does" is not correct. Fig 4b shows that increasing the number of oICMS shocks increases the response but not as effectively as the the eICMS does. This is obvious at 333Hz where the increase in the number of oICMS shocks doesn't increase the response.

Line 316 "Five pulses oICMS induced similar amplitude of EMG as five-pulses eICMS did (Fig. 4), suggesting that both oICMS and eICMS similarly activated the M1."

This is not clear in figure 4. If we look at fig 4a or figure 4b we see a clear difference between 5 pulses of oICMS and 5 pulses of eICMS. The only way the authors can say that these are similar is if they ignore the stimulation frequency, then maybe 5 shocks at 100Hz of oICMS is similar to 5 shocks 333Hz of eICMS but these comparisons will not be fair as these would ignore the effects of the frequencies.

"319 indicate that eICMS requires more pulses than oICMS to induce equivalent muscle
320 activity (Fig. 4)."
Can the authors make it clear that they relate to the threshold intensity for both type of stimulation? There is no clear statistical comparison between one shock oICMS and one shock eICMS to establish that the threshold for oICMS gave a bigger response than the threshold of eICMS in fig 4.

Fig 4C should be discussed more or at least commented more in the results. In fig 4c the increase in EMG activity is relative to the activity induced by one shock. Then the subsequent oICMS shocks do not recruit more activity. Whereas the subsequent eICMS shocks do increase the EMG activity.

Could the authors provide a justification on why they use the term “exponential” when they refer to the eICMS increase in response? Maybe they could plot the number of shocks vs the relative increase in the EMG response, and calculate the slope.

I suggest replacing this sentence
148 This phenomenon is interpreted that high-intensity oICMS induces large depolarization…
with
“The truncated action potentials that follow the stimulation could be explained by depolarization blocks induced by large depolarization in cortical neurons that last long after the cession of light illumination (up to 10 ms).”

**Author rebuttal, third version:**

Reviewer #3 (Remarks to the Author):

The revised version of the manuscript has addressed many of the concerns I expressed to the second submission. However, there are still some points that need to be clarified.

• The authors claim there was no overlap of the EGFP expression among the injections performed. However, this is difficult to judge based on the example images provided. Supplementary figure 1b (third row) shows a potential overlap between the 2 tracks indicated. It would be helpful if the authors can add some information about how the extent of one injection was defined, and how they concluded there was no overlap.

We added some information in the manuscript. (**main text, page 6**):

Each injection site was separated at least 2 mm (for the same serotype) or 3 mm (for the different serotypes) (Supplementary Fig. 1a), and there was no overlap of EGFP-positive cell bodies transduced by each serotype of viral vector in the brain sections although some EGFP-positive axons might be overlapped.

• Please provide higher magnification of the areas with presumed GFP+ terminals (panel D in supplementary figure 2). In the text, it should be mention that the areas shown (putamen, thalamus and lateral corticospinal tract) show putative axon terminals (with the available data, it cannot be firmly concluded that these are axon terminals).

Thank you for your suggestion. We added higher magnification images in **Supplementary Fig. 2d (*lower*)**.

• It is suggested that the ‘truncated shapes’ of the action potentials are due to depolarization block. This remains speculative. To back up this statement an intracellular study is referenced (ref. 20, Herman et al 2014). It would be more relevant to the current study if the authors provide references of studies showing depolarization block during extracellular recordings.

The same reference (ref. 20) reported that the absence of spikes during depolarization block was observed by extracellular recordings (Fig. 7 in ref. 20.)

• In the experiments looking at the distant effects of oICMS and eICMS the distance between the stimulation and recording sites is 2.2 mm (Supplementary fig. 5b). How was this distance chosen? Were other distances examined? It would be of great interest to know at what distance the effects disappear.

We chose 2.2 mm because both recording and stimulation electrodes were inside the forelimb area of the M1. We did not map systematically, but we could get the response up to 3.4 mm. We described as follows in the **legend of Supplementary Figure 5**:

Similar responses were obtained in other nine cases examined, where the horizontal distances between the recording and stimulation sites were 2.2 - 3.4 mm, and both were within the forelimb region of the M1.

• Based on the histology observations, it is unlikely that PV-positive interneurons were responsible for the inhibitory responses (lines 160-161). This does not rule out, however, that PV-negative inhibitory neurons may have expressed the ChR2 and played a role in the inhibitions.

Thank you for your comment. We have changed as follows (**main text, page 11**).

One possible origin of the inhibition is inhibitory cortical interneurons activated synaptically by hChR2-expressing neurons or directly by light, although PV-positive inhibitory interneurons were less labeled with EGFP (Supplementary Fig. 2b).

• Please rephrase “…were decreased more slowly…” (line 207). I believe it would be more appropriate to say that the area and peak amplitude obtained by oICMS did not show a corresponding decrease with the number of stimulus pulses.

We have changed as suggested (**main text, page 14**).

When the number of stimulus pulses was decreased, the area and peak amplitude obtained by oICMS showed a modest decrease compared to eICMS.

• How was the “Relative area of EMG” obtained? This should be explained in methods.

We added the following description (**main text, page 31**)

The relative area of EMG was defined as the ratio of the area of EMG to that evoked by single-pulse oICMS/eICMS.

• Please clarify how layer 5 neurons were identified in electrophysiological recordings (lines 265, 742 and 748). If the neurons were putatively identified as layer 5 neurons (as suggested in lines 742 and 748) this should be made clear throughout the manuscript.

In most cases, it is described such as “ probably layer 5”.

We added “putative” before “layer 5” (**main text, pages 26**).

• I suggest to use only ‘light intensity’ instead of ‘laser light intensity’ throughout the manuscript. ‘Laser light’ seems redundant.

I have changes as suggested except some cases, which we would like emphasize laser.

• In Discussion, lines 285 through 286 (list of other excitatory opsins) seems irrelevant. I suggest removing this part.

We have deleted as suggested.

• The wording “laser light sheds layer 5 from the side and can activate layer 5 pyramidal neurons over a wide area” (lines 326-237) is awkward. Can this be reworded?

We changed as follows (**main text, page 22**):

When the optrode is inserted into the anterior bank of the central sulcus, light can activate layer 5 pyramidal neurons over a wide area.

• In Discussion the authors state that “during high-frequency optical stimulation, stimulus pulses often fail in inducing action potentials: (lines 335 and 336). This statement contradicts the results, “Figure 2b also shows that action potentials followed each stimulus pulse at 200 Hz (Fig. 2b, 15 mW corresponding to 1910 mW/mm2, 2 ms, 200 Hz, 10 pulses” (lines 163-164). Please clarify.

This description is based on previous works. We clarified as follows (**main text, page 22**):

Actually, during high-frequency optical stimulation, more than 100 Hz, stimulus pulses often fail in inducing action potentials in previous studies ^39, 40^.

• Line 60 “stronger light”. Please clarify this means stronger light intensity than in previous studies.

We changed as follows (**main text, page 5**):

using higher intensity light than previous studies

• In supplementary figure 1b, please indicate from which area of the low magnification image was the high magnification image obtained.
Thank you for your suggestion. We added squares in **Supplementary Fig. 1b** to indicate which area are enlarged.

Reviewer #5 (Remarks to the Author):

The authors have changed their manuscript and figures and have addressed many of the concerns. This has made their article stronger. I still think this is a method paper that demonstrates for the first time, that optogenetic stimulation of motor cortex in non-human primate can be achieved with the use of a specific virus. This can be of a great interest to the scientific community. The spiking activity in fig 2 is worth documenting by itself as this kind of responses are not visible with eICMS because of the large artefact that electrical stimulation will induce. This is one advantage of the oICMS that is worth discussing. However the characterisation of the cells recruited by the oICMS to achieve this motor responses has not been fully considered specially regarding the corticospinal tract neurons. Also I have concerns regarding the interpretation of the results shown in figure 4.

Can the image on the figure2d in the supplementary data for the corticospinal tract be zoomed out to see that it is the lateral corticospinal tract in the spinal cord and zoomed in so we can see clearly the expression of the EGFP?

We added lower and higher magnification images of the spinal cord in **Supplementary Fig. 2d (*upper and lower* in the *middle*)**.

The expression of the virus in the tract will demonstrate that the motor effect induced by oICMS in M1 is due partly to direct stimulation of the corticospinal tract, even though this link is hard to establish because three different viruses were injected in the same animal which makes it difficult to distinguish which virus has transfected the corticospinal neurons. 
Thank you for your comment. We agree that it is difficult to distinguish which viral vector transduced the expression of EGFP in the corticospinal tract in this experiment. If we can develop the viral vector which can be expressed ChR2/EGFP specifically in projection neurons of the M1 and can demonstrate the motor effect induced by oICMS in monkeys, we are going to observe the spinal cord in detail in the near future.

The statement that "repetitive oICMS induces muscle activity effectively as repetitive eICMS does" is not correct. Fig 4b shows that increasing the number of oICMS shocks increases the response but not as effectively as the the eICMS does. This is obvious at 333Hz where the increase in the number of oICMS shocks doesn't increase the response.

We have changed as suggested (**main text, page 15**):

These results suggest that repetitive oICMS as well as eICMS induces muscle activity more effectively than single-pulse oICMS and eICMS.

Line 316 "Five pulses oICMS induced similar amplitude of EMG as five-pulses eICMS did (Fig. 4), suggesting that both oICMS and eICMS similarly activated the M1."
This is not clear in figure 4. If we look at fig 4a or figure 4b we see a clear difference between 5 pulses of oICMS and 5 pulses of eICMS. The only way the authors can say that these are similar is if they ignore the stimulation frequency, then maybe 5 shocks at 100Hz of oICMS is similar to 5 shocks 333Hz of eICMS but these comparisons will not be fair as these would ignore the effects of the frequencies.

We have deleted this sentence.

"319 indicate that eICMS requires more pulses than oICMS to induce equivalent muscle
320 activity (Fig. 4)."
Can the authors make it clear that they relate to the threshold intensity for both type of stimulation? There is no clear statistical comparison between one shock oICMS and one shock eICMS to establish that the threshold for oICMS gave a bigger response than the threshold of eICMS in fig 4. 
We have deleted above description from the text. Instead, we simply described as follows (**main text, page 21**).

Both oICMS and eICMS can induce neuronal and muscle activity effectively (Figs. 3, 4, Supplementary Fig. 5), although different population would be stimulated.

Fig 4C should be discussed more or at least commented more in the results. In fig 4c the increase in EMG activity is relative to the activity induced by one shock. Then the subsequent oICMS shocks do not recruit more activity. Whereas the subsequent eICMS shocks do increase the EMG activity.

We described as follows (**main text, page 16**):

The different effects by oICMS and eICMS were also observed in the relative area of EMG and the semilog plots of the area of EMG evoked by repetitive oICMS/eICMS (Fig. 4c, Supplementary Fig. 6a). The relative area of EMG evoked by oICMS increased as the numbers of stimulus pulses increased at 100 Hz, but did not at 200 and 300 Hz (Fig. 4c). On the other hand, the relative area of EMG by eICMS increased as the number of stimulus pulses increased at all frequencies. The semilog plots indicate that the area of EMG evoked by eICMS increased more rapidly than that by oICMS as the numbers of stimulus pulses increased in a nearly exponential manner (Supplementary Fig. 6a). These results suggest that oICMS shows less temporal summation than eICMS. The peak amplitude of EMG showed similar tendency (Supplementary Fig. 6b).

Could the authors provide a justification on why they use the term “exponential” when they refer to the eICMS increase in response? Maybe they could plot the number of shocks vs the relative increase in the EMG response, and calculate the slope.

We plotted in **Supplementary Figure 6a**, and described as follows in the **figure legend of Supplementary Figure 6**:

The logarithm of the area of EMG in each experiment is plotted against numbers of stimulus pulses in scatter graphs. Different colors indicate different frequencies (single pulse, 100, 200, and 333 Hz). Liner regression lines, regression equations, and coefficient of determination (R^2^) are shown. eICMS exhibits steeper slopes of regression lines and larger R^2^ than oICMS, indicating that the area of EMG evoked by eICMS increased more rapidly than that by oICMS as the numbers of stimulus pulses increased and could be approximated by an exponential.

I suggest replacing this sentence
148 This phenomenon is interpreted that high-intensity oICMS induces large depolarization…
with
“The truncated action potentials that follow the stimulation could be explained by depolarization blocks induced by large depolarization in cortical neurons that last long after the cession of light illumination (up to 10 ms).”

We changed as suggested (**main text, page 10**):

The truncated action potentials that follow the stimulation could be explained by depolarization blocks induced by large depolarization in cortical neurons that last long after the cession of light illumination (up to 10 ms)^20^

**Reviewer comments, fourth version:**

Reviewer #3

Remarks to the Author:
The new revised version of the manuscript by Watanabe et al. has addressed most of the remaining concerns. I only have a few more minor suggestions to make.
- I do not think that the authors can completely rule out overlap of EGFP expression among the injections. They should simply point this out as a technical limitation of the study.
- As previously mentioned, I believe the use of the term “laser light” is inappropriate (lines 146 and 305). Laser is an acronym for ‘light amplification by stimulated emission of radiation’, and thus ‘laser light’ is a pleonasm. I suggest to replace by ‘laser’.
